# Recombinant production of *Paenibacillus wynnii β*-galactosidase with *Komagataella phaffii*

**DOI:** 10.1186/s12934-024-02544-5

**Published:** 2024-10-05

**Authors:** Anna Bechtel, Ines Seitl, Eva Pross, Frank Hetzel, Mario Keutgen, Lutz Fischer

**Affiliations:** Institute of Food Science and Biotechnology, Department of Biotechnology and Enzyme Science, University of Hohenheim, Garbenstr. 25, 70599 Stuttgart, Germany

**Keywords:** *Komagataella phaffii*, *Paenibacillus wynnii*, *β*-Galactosidase, Lactose hydrolysis

## Abstract

**Background:**

The *β-*galactosidase from *Paenibacillus wynnii* (*β-*gal-Pw) is a promising candidate for lactose hydrolysis in milk and dairy products, as it has a higher affinity for the substrate lactose (low *K*_M_ value) compared to industrially used *β-*galactosidases and is not inhibited by the hydrolysis-generated product D-galactose. However, *β-*gal-Pw must firstly be produced cost-effectively for any potential industrial application. Accordingly, the yeast *Komagataella phaffii* was chosen to investigate its feasibility to recombinantly produce *β-*gal-Pw since it is approved for the regulated production of food enzymes. The aim of this study was to find the most suitable way to produce the *β-*gal-Pw in *K. phaffii* either extracellularly or intracellularly.

**Results:**

Firstly, 11 different signal peptides were tested for extracellular production of *β*-gal-Pw by *K. phaffii* under the control of the constitutive GAP promoter. None of the signal peptides resulted in a secretion of *β-*gal-Pw, indicating problems within the secretory pathway of this enzyme. Therefore, intracellular *β-*gal-Pw production was investigated using the GAP or methanol-inducible AOX1 promoter. A four-fold higher volumetric *β-*galactosidase activity of 7537 ± 66 µkat_*o*NPGal_/L_culture_ was achieved by the *K. phaffii* clone 27 using the AOX1 promoter in fed-batch bioreactor cultivations, compared to the clone 5 using the GAP promoter. However, a two-fold higher specific productivity of 3.14 ± 0.05 µkat_*o*NPGal_/g_DCW_/h was achieved when using the GAP promoter for *β-*gal-Pw production compared to the AOX1 promoter. After partial purification, a *β-*gal-Pw enzyme preparation with a total *β-*galactosidase activity of 3082 ± 98 µkat_*o*NPGal_ was obtained from 1 L of recombinant *K. phaffii* culture (using AOX1 promoter).

**Conclusion:**

This study showed that the *β-*gal-Pw was produced intracellularly by *K. phaffii*, but the secretion was not achieved with the signal peptides chosen. Nevertheless, a straightforward approach to improve the intracellular *β-*gal-Pw production with *K. phaffii* by using either the GAP or AOX1 promoter in bioreactor cultivations was demonstrated, offering insights into alternative production methods for this enzyme.

**Supplementary Information:**

The online version contains supplementary material available at 10.1186/s12934-024-02544-5.

## Background

*β-*Galactosidases (EC 3.2.1.23) catalyze the hydrolysis of terminal non-reducing *β-*D-galactose residues in *β-*D-galactosides by breaking a glycosidic bond. Accordingly, *β-*galactosidases are able to cleave lactose into the monosaccharides D-glucose and D-galactose, and are, therefore, mainly used for lactose hydrolysis in milk or dairy products [1–4]. Lactose intolerance is a widespread problem among humans, therefore, there is a great demand for lactose-free products [5, 6]. In addition, lactose hydrolysis can prevent lactose crystallization, which otherwise could lead to a mealy, sandy or gritty texture of the product. The sweetness of the product can also be increased due to the formation of D-glucose and D-galactose [7]. Besides the hydrolysis reaction, *β-*galactosidases can catalyze the transgalactosylation reaction by transferring galactose, which is bound in the active center as an intermediate during lactose cleavage, onto a nucleophilic hydroxyl group of another lactose, resulting in galactooligosaccharides (GOS). The GOS are prebiotics stimulating the growth of beneficial bacteria, such as bifidobacteria in the human intestine [8]. Therefore, GOS production is important for the food industry and they are applied as functional food mainly in infant formula and toddler nutrition [9, 10].

The kinetic properties of *β-*galactosidases used in the food industry are still not optimal for the lactose hydrolyzing process. This is due to the inhibition by the hydrolysis-generated product D-galactose and a low affinity to the substrate lactose [1, 4, 6, 11], which is currently compensated by using high amounts of cheap *β-*galactosidases. By contrast, the *β-*galactosidase from *Paenibacillus wynnii* (*β-*gal-Pw; EC 3.2.1.23) possesses a lower *K*_M_ value of 0.63 mM for lactose than known commercial *β-*galactosidases from *Aspergillus oryzae* (*K*_M, lactose_: ∼ 52 mM), *Bacillus circulans* (*K*_M, lactose_: ∼ 16 mM) or *Kluyveromyces lactis* (*K*_M, lactose_: ∼ 31 mM) [12, 13]. If the structural properties are compared, the commercially used *β-*galactosidases mentioned are active as monomers, with the exception of the *β-*galactosidase from *K. lactis*, which is active as a tetramer [14–16]. For *β-*gal-Pw also a monomeric structure was observed by size exclusion chromatography in a previous study [17]. A common feature of all these *β-*galactosidases is their high molecular weight for the monomeric form ranging from 110 kDa (*A. oryzae β-*gal) to 190 kDa (*B. circulans β-*gal). Another advantage is, that *β-*gal-Pw is not inhibited by D-galactose like the commercially used *β-*galactosidases, thus, leading to a more effective hydrolysis of lactose [12, 13]. Due to the mentioned characteristics, *β-*gal-Pw is of potential interest to the dairy industry. *β-*gal-Pw was first produced recombinantly in *E. coli* BL21(DE3) [13], however, an alternative producer species is required for application in the food industry. Senger et al., therefore, investigated the extracellular production of the *β-*gal-Pw with *Bacillus subtilis* SCK6, but a 17-fold lower *β-*galactosidase activity per liter of culture was achieved compared to the intracellular production in *E. coli* BL21(DE3) [13, 17].

The methylotrophic yeast *Komagataella phaffii* (formerly known as *Pichia pastoris*) was already being used for the high-level recombinant production of several industrial enzymes [18, 19]. Additionally, *K. phaffii* possesses the European Food Safety Authority’s ‘qualified presumption of safety’ (QPS) status and the Food and Drug Administration’s ‘generally recognized as safe’ (GRAS) status and is, thus, allowed for the production of enzymes used in the food industry [20–22]. Another advantage of this yeast is that high cell densities of more than 100 g dry cell weight/L can be achieved on inexpensive culture media [23, 24]. Different culture conditions and feed strategies were used for the cultivation of *K. phaffii*, depending on the promoter used for recombinant protein expression [25, 26]. Two of the promoters mainly used are the inducible alcohol oxidase 1 (P_AOX1_) and the constitutive GAP promoter (P_GAP_) [27–29]. P_AOX1_ is repressed in the presence of glucose and glycerol and strongly induced by methanol [27, 30]. However, the strength of the constitutive P_GAP_ promotor depends on the carbon source and is highest when cells are grown on glucose, followed by glycerol and methanol [28]. The specific productivity *q*_p_ of *K. phaffii* clones using P_AOX1_ is often higher when the specific growth rate µ is below the maximum specific growth rate µ_max_, while the *q*_p_ of clones using P_GAP_ is often higher when the µ is near to µ_max_ [26]. Therefore, promoter-specific cultivation conditions should be chosen to achieve the highest possible yield with the respective promoter.

*β-*Galactosidases from various organisms have already been successfully produced in *K. phaffii* [31–34]. Up to now, the highest yield of a recombinant *β-*galactosidase (*Paecilomyces aerugineus*) secreted with *K. phaffii* was 9500 kU_*o*NPGal_/L_culture_ (158 mkat_*o*NPGal_/L_culture_) and published uniquely in 2011 [32]. The secretion of the desired enzyme in *K. phaffii* has the advantage of simple downstream processing because *K. phaffii* secretes only a low number of endogenous proteins [35].

In this study, we investigated the high-level production of *β-*gal-Pw in *K. phaffii*, by testing different signal peptides for extracellular production and two different promoters.

## Methods

### Chemicals, enzymes and kits

The chemicals used in this study were of analytical grade and, if not specifically mentioned, were purchased from Carl Roth (Karlsruhe, Germany), Sigma Aldrich (St. Louis, USA) and Fisher Scientific (Hampton, USA). Peptone (from casein, enzymatic digest) was bought from Sigma-Aldrich. The antibiotics zeocin and G418 were purchased from InvivoGen (San Diego, USA). Kanamycin sulfate, chloramphenicol and bovine serum albumin (modified Cohn Fraction V, pH 5.2) were acquired from Serva electrophoresis GmbH (Heidelberg, Germany). The MoClo *Pichia* Toolkit (provided by Volker Sieber; Addgene kit #1000000108) and MoClo Yeast Toolkit (provided by John Dueber; Addgene kit #1000000061) were obtained from Addgene (Watertown, USA) [36, 37]. Restriction enzymes and the Q5^®^ High-Fidelity DNA Polymerase was procured from New England Biolabs GmbH (Frankfurt am Main, Germany). TaKaRa Ex Taq^®^ DNA Polymerase was purchased from TaKaRa Bio Inc. (Kusatsu, Japan). The GeneRuler 1 kb Plus DNA Ladder was purchased from Thermo Fisher Scientific (Waltham, Massachusetts, USA). Roti^®^-Phenol/Chloroform/isoamyl alcohol and X-Gal (5-bromo-4-chloro-3-indolyl-*β*-D-galactopyranoside) was bought from Carl Roth (Karlsruhe, Germany). Antifoam 204 (A6426) was acquired from Sigma-Aldrich (St. Louis, USA). Glucose rapidtest quantofix was purchased from Macherey-Nagel (Düren, Germany) and the glycerol assay kit from Megazyme (Bray, County Wicklow, Ireland). The Precision Plus Protein™ unstained protein standard (10–250 kDa) was obtained from Bio-Rad Laboratories GmbH (Feldkirchen, Germany). Trypsin (proteomics grade) was procured from Roche (Basel, Switzerland). The commercial *β*-galactosidase preparations Saphera 2600L and Lactozym Pure 6500L (Novozymes, Bagsværd, Denmark), Maxilact Super (DSM, Heerlen, Netherlands), GODO-YNL2 (GODO SHUSEI Co., Ltd., Chiba, Japan) and Dairyzym Y 50 L (SternEnzym GmbH & Co. KG, Ahrensburg, Germany) were used for comparison with the *β*-gal-Pw preparation.

### Wildtype strains and media

*Escherichia coli* XL1-blue was used for the plasmid propagation and cloning procedures and grown in lysogeny broth (LB) medium [38] at 37 °C containing the appropriate antibiotic (50 µg/mL kanamycin or 40 µg/mL chloramphenicol).

*Komagataella phaffii* ATCC 76273 (CBS7435 or NRRL Y-11430) was obtained from the American Type Culture Collection and grown at 30 °C in different media depending on the experiment. *K. phaffii* transformants were selected on YPDS agar plates prepared with 15 g/L agar-agar [38] containing the appropriate antibiotic (100 µg/mL zeocin or 500 µg/mL G418). *K. phaffii* transformants investigated for *β-*gal-Pw secretion were additionally restreaked on BMD_X−Gal_ agar plates containing the components of BMD medium (with 20 g/L glucose) [39], 15 g/L agar-agar and 80 µg/mL X-Gal. Microscale screening experiments and shake flask cultivations of recombinant *K. phaffii* strains were done in YPD [38] or BMGY and BMMY medium [39]. Precultures for *K. phaffii* bioreactor cultivations were done in BMD (with 100 mM potassium phosphate buffer pH 5 or 6) or BMG (with 100 mM potassium phosphate buffer pH 5) medium [39]. Bioreactor cultivations were carried out in BSM medium, starting the batch phase with 40 g/L of either glycerol or glucose, containing 4.35 mL/L PTM_1_ trace salts solution [40].

### Plasmid construction

Plasmids and primers used in this study are listed in the Additional file 1: Tables S1–S3. Plasmids were constructed using the MoClo *Pichia* (PTK) and MoClo Yeast Toolkit (YTK) [36, 37]. New sequences were included in the MoClo PTK by cloning the sequences into the pYTK001 plasmid (entry vector) to construct so-called part plasmids, as described by Lee et al. [37]. Newly constructed part plasmids were verified by sequencing. The signal peptide sequence of the endo-1,3(4)-*β*-glucanase (UniProt ID: C4QW71) was firstly amplified from the *K. phaffii* genome by Q5^®^ High-Fidelity DNA Polymerase using the C4QW71-fw and C4QW71-rev primers and then cloned into pYTK001 generating the part plasmid pPTK022. The *β-gal-Pw* gene (GenBank: MP751470.1) was codon optimized for *K. phaffii* and synthesized by Invitrogen (Thermo Fisher Scientific) (Additional file 1: Fig. S1). The *β-gal-Pw* gene was amplified using the primers *β-*gal-Pw-3b-fw and *β*-gal-Pw-rev and cloned into pYTK001 generating the part plasmid pPTK023 for extracellular production. The *β-gal-Pw* gene was amplified using the primers *β-*gal-Pw-3-fw and *β*-gal-Pw-rev and cloned into pYTK001 generating the part plasmid pPTK024 for intracellular production.

Cassette plasmids containing the *β*-gal-Pw expression cassettes were constructed as described previously [37] and correct assembly was verified by restriction digestion. The vector backbone of all cassette plasmids was the same, therefore, a GFP dropout plasmid consisting of a GFP expression cassette dropout, ZeoR, *attB*, KanR-CoIE1 and two assembly connectors was constructed. The GFP expression cassette was replaced with the respective *β*-gal-Pw expression cassette to construct cassette plasmids for extracellular or intracellular *β-*gal-Pw production.

### Construction of recombinant *K. phaffii* strains

Cassette plasmids containing the *β*-gal-Pw expression cassettes were integrated into either the TRP2, GAP or AOX1 locus in the *K. phaffii* genome. Integration into the TRP2 locus was done by a recombinase-based method, according to Perez-Pinera et al. [41]. The BxbI recombinase expression vector PP43 (Addgene plasmid #78953) and the PP74 vector (Addgene plasmid #78944), which contains the *attP* site for BxbI recombinase, were purchased by Addgene (provided by Timothy Lu) [41]. The PP74 vector was linearized by *Kpn*I and integrated into the TRP2 locus by homologous recombination. Integration was verified using the primers TRP2-fw and TRP2-rev (Additional file 1: Table S3). Subsequently, cassette plasmids were simultaneously transformed with the BxbI recombinase expression vector, as described by Perez-Pinera et al. [41]. The BxbI recombinase catalyzes site-specific recombination between the *attP* (encoded on PP74 integrated in TRP2 locus) and *attB* sites (encoded on each cassette plasmid), resulting in the integration of the respective cassette plasmid into the TRP2 locus. Integration of cassette plasmids into the GAP or AOX1 locus was realized by homologous recombination. The cassette plasmids were linearized using *Avr*II (when integrated into the GAP locus) or *Pme*I (when integrated into the AOX1 locus) before the transformation of *K. phaffii*. Transformation was generally carried out according to Perez-Pinera et al. [41]. Electroporation was done with the MicroPulser™ (Bio-Rad, Hercules, USA) using the Sc2 program. Transformants were selected on YPDS agar plates containing the appropriate antibiotic (zeocin or G418).

Cassette plasmids for extracellular *β-*gal-Pw production were integrated into the *K. phaffii* genome using the BxbI recombinase-based method. Genomic integration of these cassette plasmids was verified by restreaking the colonies on BMD_X−Gal_ agar plates.

Cassette plasmids for intracellular *β*-gal-Pw production were integrated either into the TRP2 locus by the BxbI recombinase-based method or into the GAP or AOX1 locus by homologous recombination. Integration of the cassette plasmids into the respective locus was verified by genomic DNA PCR using TaKaRa Ex Taq^®^ DNA Polymerase. The genomic DNA of the *K. phaffii* transformants was isolated based on Harju et al. [42]. However, instead of freezing and thawing, 0.3 g glass beads (Ø 0.5–0.7 mm) were added for cell disruption and Roti^®^-Phenol/Chloroform/isoamyl alcohol was added instead of chloroform before vortexing for 3 min. An amount of 200–250 ng DNA was used as a template for the PCR reaction. Primer pairs with one primer binding to the *K. phaffii* genome upstream of the integrated cassette plasmid and one binding within the integrated cassette plasmid were used. The primer pairs TRP2-fw and ConLS-rev (TRP2 locus), GAP-fw and tAOX1-rev (GAP locus), and AOX1-fw and 5’-*β*-gal-Pw-rev (AOX1 locus) were used for the verification of the cassette plasmid integration into the respective locus (Additional file 1: Table S3).

### Microscale screening

Recombinant *K. phaffii* strains with integrated cassette plasmids for *β*-gal-Pw secretion (P_GAP_ used for expression) were cultivated in honeycomb 2 plates (Fisher Scientific, Waltham, USA) in biological duplicates. Cultivation was rendered in 250 µL YPD medium per well. Each well was inoculated with a single colony using a sterile toothpick. Cultivation was done at 30 °C and 600 rpm on a Titramax 101 shaker (Heidolph Instruments GmbH & Co. KG, Schwabach, Germany). After 24 h, the honeycomb plates were centrifuged (2000 x g, 15 min, 4 °C) and culture supernatants were investigated for *β-*galactosidase activity. Up to 10 *K. phaffii* clones of each construct (differing in signal peptide) were investigated.

Recombinant *K. phaffii* strains with integrated cassette plasmids for intracellular *β*-gal-Pw production were cultivated in deep well plates 96/2000 µL (Eppendorf AG, Hamburg, Germany) in biological triplicates. Cultivation was done in YPD medium if P_GAP_ was used for *β-*gal-Pw production and BMGY/BMMY medium if P_AOX1_ was used. Precultures were done in sterile tubes using 3 mL YPD or BMGY medium. The precultures were incubated at 30 °C and 180 rpm overnight. Precultures were diluted to an OD_600nm_ of 0.5 using the respective medium. The main cultivation was realized in deep well plates using a 500 µL working volume. An amount of 450 µL fresh YPD or BMGY medium was filled into each well and inoculated with 50 µL of diluted preculture (initial OD_600nm_ of 0.05). The plate was covered by a Breathe-Easier sealing membrane (Sigma-Aldrich, St. Louis, USA) and incubated at 30 °C and 900 rpm on a Titramax 101 shaker. The cultivation in YPD medium was done for 48 h. The cultivation in BMGY medium was done for 24 h. Afterwards, the deep well plate was centrifuged (2000 x g, 15 min, 4 °C), the supernatants were discarded, the cell pellets were resuspended in 500 µL BMMY medium and the cultivation was continued for another 48 h. After 24 h cultivation in BMMY medium 0.5% (v/v), methanol was added to maintain induction conditions. Cells were harvested by centrifugation (2000 x g, 15 min, 4 °C) and disrupted in a microtiter plate to determine the intracellular *β*-galactosidase activity (see below).

### Shake flask cultivations

Shake flask cultivations of *K. phaffii* clones with the *β-gal-Pw* gene fused to various signal peptides under the control of P_GAP_ were done at 30 °C for 76 h using YPD medium. Recombinant *K. phaffii* clones were cultivated in a working volume of 100 mL in 500 mL baffled shake flasks (110 rpm, 76 h), each inoculated with 10% (v/v) preculture (done in sterile tube at 180 rpm, 20 h). Two samples were taken per day to determine the optical density (OD_600nm_) and extracellular *β*-galactosidase activity. Intracellular *β-*galactosidase activity was determined 48 h after inoculation. Regarding *β*-galactosidase activity determination, 2 mL of the cell culture was centrifuged (13,000 x g, 5 min, 4 °C), the cell-free supernatant was used to determine the extracellular *β*-galactosidase activity and the cell pellet was disrupted to determine the intracellular *β*-galactosidase activity (see below).

### Fed-batch bioreactor cultivations

Fed-batch bioreactor cultivations were fulfilled based on the “*Pichia* Fermentation Process Guidelines” from Invitrogen [40]. The *K. phaffii* clone using the Killer*-α*MF∆ signal peptide for *β*-gal-Pw secretion was cultivated in the Labfors 5 bioreactor (Infors HT, Bottmingen, Switzerland) with a total vessel volume of 7.5 L. Recombinant *K. phaffii* clones producing *β-*gal-Pw intracellularly under the control of P_GAP_ or P_AOX1_ were cultivated in Multifors 2 bioreactors (Infors HT, Bottmingen, Switzerland) with a total vessel volume of 1.4 L. Cultivations were done at 30 °C and pH 5 (intracellular production) or pH 6 (extracellular production).

Precultures were done in BMD (P_GAP_) or BMG (P_AOX1_) medium. Firstly, the preculture was inoculated with a single colony, and the following precultures were inoculated with 10% (v/v) of the respective working volume. Each preculture was incubated for 24 h. The main culture in the bioreactor was inoculated with 10% (v/v) initial fermentation volume. The initial fermentation volume for the Multifors 2 bioreactors was 500 mL and 3 L for the Labfors 5 bioreactor. The batch phase in the bioreactor was realized in BSM medium containing a PTM_1_ trace salts solution using either glucose (P_GAP_) or glycerol (P_AOX1_) as the carbon source. Antifoam 204 was added once before inoculation (50 µL/L), and then only as needed (the amount was kept to a minimum). The pH was kept constant with 25% ammonium hydroxide and 2 M H_3_PO_4_. The bioreactors were aerated with 1–2 vvm and supplemental oxygen was added as needed during the fed-batch phase to maintain the pO_2_ above 20%. The fed-batch phase was started when glucose or glycerol was completely consumed, indicated by a peak in the pO_2_ and verified by glucose test strips (glucose rapidtest quantofix) and a glycerol assay kit, respectively. If P_GAP_ was used for *β-*gal-Pw production, an exponential glucose feed using a 400 g/L glucose solution containing 12 mL/L PTM_1_ trace salts solution was applied. Firstly, the µ_max_ of the strain used was determined based on the dry cell weight values obtained during a previous batch bioreactor cultivation under conditions described above. A µ ≤ µ_max_ was chosen for the fed-batch phase. The exponential feed rate (F(t)) and initial feed rate (F_0_) were calculated, as described previously by Looser et al. [26]. If P_AOX1_ was used for the *β-*gal-Pw production, a stepwise increasing methanol feed was applied. Thereby, 100% methanol containing 12 mL/L PTM_1_ trace salts solution was used as the feed solution. The feed rate 1 was 1.8 mL/h, feed rate 2 was 3.64 mL/h and feed rate 3 was 5.45 mL/h. The concentration of methanol in the bioreactor was determined by gas chromatography to prevent methanol accumulation.

Samples were taken during cultivation to determine the OD_600nm_, the wet and dry cell weight, as well as the *β*-galactosidase activity. Cells obtained after bioreactor cultivations were harvested by centrifugation (6,000 x g, 15 min, 4 °C). The cells were washed with saline (0.9% (w/v) NaCl) and finally centrifuged at 8,000 x g and 4 °C for 15 min, before it was stored at -20 °C until further processed.

### Determination of methanol concentration by gas chromatography

The methanol concentration in cell-free culture supernatants was determined by gas chromatography using the GC-2010 Plus System (Shimadzu; Kyoto, Japan) equipped with an AOC-20i autoinjector and a flame ionizing detector. The chromatographic separation was done on a Zebron ZB-1701 column (L = 30 m; ID = 0.25 mm; FT = 0.25 μm) from Phenomenex (Torrance, California, USA). A sample volume of 1 µL was injected in split mode (split ratio: 125). The detector and injector temperatures were set to 330 °C and 200 °C, respectively. The column oven temperature was initially set to 30 °C (held for 4 min) and then elevated to 250 °C at a rate of 25 °C/min and maintained for 10 min. Helium was used as the carrier gas at a flow rate of 0.74 mL/min. The data from gas chromatography runs were analyzed using the LabSolutions™ software from Shimadzu (Kyoto, Japan). A calibration curve using 0.05–5% (v/v) methanol in H_2_O_dd_ was done to evaluate the data.

### Cell disruption

After cultivation of recombinant *K. phaffii* strains in deep well plates, shake flasks and bioreactors, cell disruption was done in different vessels. In general, cooled (∼ 4 °C) 100 mM potassium phosphate buffer containing 5 mM MgCl_2_ and 1 mM PMSF (pH 6.75) was used for the cell disruption.

Each cell pellet obtained after deep well plate cultivation was resuspended in 200 µL buffer and subsequently transferred to a microtiter plate well (microtitration plates ROTILABO^®^ F-profile; Carl Roth, Karlsruhe, Germany) containing 0.2 g glass beads (Ø 0.5–0.7 mm). The microtiter plate was sealed using a sealing mat (piercable TPE capmat; Micronic, Lelystad, Netherlands). Cell disruption was done using the TissueLyser II (Qiagen, Hilden, Germany) at a frequency of 30/s for 15 min. Afterwards, the microtiter plate was centrifuged (2,000 x g, 15 min, 4 °C) and the cell-free supernatant was used for the *β-*galactosidase activity determination (see below).

Cell pellets obtained from the shake flask cultivations were resuspended in 1.5 mL buffer, while 20% (w/v) cell suspensions in buffer were prepared with cell pellets obtained from the bioreactor cultivations. An amount of 1.5 mL of the cell suspension was added to 1.5 g glass beads (Ø 0.75 mm) in a 2 mL Eppendorf tube. The cells were disrupted using the TissueLyser II (Qiagen, Hilden, Germany) at a frequency of 30/s for 15 min. Afterwards, the samples were centrifuged (13,000 x g, 10 min, 4 °C) and the cell-free supernatant was used for the *β*-galactosidase activity and protein determination (see below).

Regarding the large-scale production of a *β*-gal-Pw preparation, 450 g of *K. phaffii* bio wet mass (obtained from ∼ 1 L cell culture of bioreactor cultivation using P_AOX1_ for *β*-gal-Pw production) was disrupted in a 600 mL (total volume) vessel using the DYNO^®^-MILL KDL A (Willy A. Bachofen GmbH, Niedderau, Germany). A 40% (w/v) cell suspension was prepared in buffer. The vessel was filled with 80% (v/v) glass beads (Ø 0.75 mm) and the cell disruption was carried out in continuous mode at 2,000 rpm by pumping the cell suspension through the vessel with a residence time of about 8 min. The sample was cooled at 5 °C during the disruption. After disruption, the cell debris was removed by centrifugation (24,000 x g, 1 h, 4 °C) and the supernatant used for the *β*-gal-Pw purification.

### Purification of *β*-gal-Pw and formulation of an enzyme preparation

After the disruption of recombinant *K. phaffii* cells using the DYNO^®^-MILL, the cell-free extract obtained was used for the purification of the *β*-gal-Pw. A 4 M (NH_4_)_2_SO_4_ solution was added dropwise to the cell-free extract and stirred on ice until a (NH_4_)_2_SO_4_ saturation of 20% (250 mL of 4 M (NH_4_)_2_SO_4_ per liter cell free extract) was reached, according to Scopes [43]. Equilibration was done overnight on ice with stirring. Thereafter, the solution was centrifuged (24,000 x g, 1 h, 4 °C) and the pH of the supernatant obtained was adjusted to pH 6.75 using 5 M NaOH. This sample was then used for the subsequent hydrophobic interaction chromatography (HIC) using an ÄKTA FPLC system (GE Healthcare, Chicago, Illinois, USA). Accordingly, an empty column (ID = 60 mm, L = 33 cm, p_max_ = 20 bar) from Latek Labortechnik GmbH (Eppelheim, Germany) was packed with Toyopearl Phenyl-650 M resin (Tosoh Bioscience, Tokyo, Japan). The sample was applied to the column (column volume (CV) = 400 mL) at a flow rate of 25 mL/min. Unbound proteins were washed out with binding buffer (100 mM potassium phosphate buffer containing 0.86 M (NH_4_)_2_SO_4_; pH 6.75) at a flow rate of 50 mL/min for 4 CV. The flow rate of 50 mL/min was also used for the remaining purification. Bound proteins were eluted from the column using step elution. The first elution step was done for 5 CV using 20% elution buffer (100 mM potassium phosphate buffer containing 5 mM MgCl_2_; pH 6.75). Afterwards, *β*-gal-Pw was eluted using 100% elution buffer for 5 CV. Fractionation during purification was done manually in up to 2 L bottles. Regarding the determination of the protein concentration and *β*-galactosidase activity, 2.5 mL of each sample was desalted against 100 mM potassium phosphate buffer containing 5 mM MgCl_2_ (pH 6.75) using PD-10 columns (GE Healthcare, Chicago, USA). Active *β*-gal fractions were pooled and formulated into an enzyme preparation. The pooled active fractions were firstly concentrated approximately four-fold using a Vivaflow 200 crossflow cassette (MWCO 10 kDa; Sartorius, Göttingen, Germany) at 4 °C. Subsequently, the concentrate was desalted (five-fold) via diafiltration, using the Vivaflow 200 cassette, to 100 mM potassium phosphate buffer containing 5 mM MgCl_2_ and 150 mM NaCl (pH 6.75). Afterwards, 50% (/v) glycerol was added to obtain the final enzyme preparation.

### Protein analysis

Before analysis, samples were initially desalted against 100 mM potassium phosphate buffer containing 5 mM MgCl_2_ (pH 6.75) using PD-10 columns (GE Healthcare, Chicago, USA). The protein concentration of enzyme samples was determined according to the method of Bradford, using bovine serum albumin as a standard [44]. Samples were also analyzed by sodium dodecyl sulfate (SDS)-polyacrylamide gel electrophoresis (PAGE) on a 8% sparating gel [45]. An amount of 5 µg protein was loaded onto each lane of the gel. The Precision Plus Protein™ unstained protein standard (10–250 kDa) was used for molecular mass determination. Protein bands were stained with Coomassie Brilliant Blue G-250 [46]. The cell-free culture supernatant was concentrated 160-fold by trichloroacetic acid precipitation before loaded onto the gel for the analysis of extracellular *β*-gal-Pw. Proteins were precipitated with 20% (w/v) trichloroacetic acid, incubated at 4 °C overnight, washed with ice-cold acetone, air-dried and resuspended in 1 x SDS sample buffer (0.02% (w/v) Tri-HCl, 6% (w/v) glyerol, 0.1% (w/v) brophenol blue, 4% (w/v) SDS and 2% (w/v) *β*-mercaptoethanol) before loading into the gel pocket.

### Determination of the *β*-galactosidase activity

The *β*-galactosidase activity was determined according to Erich et al. [4], using *ortho*-nitrophenol-*β*-D-galactopyranoside (*o*NPGal) as substrate. After separate preincubation (37 °C, 800 rpm, 10 min), 80 µL of the activity buffer (100 mM potassium phosphate, 5 mM MgCl_2_, pH 6.75) was mixed with 100 µL substate solution (50 mM *o*NPGal dissolved in activity buffer) and 20 µL enzyme solution (desalted against activity buffer, if necessary) was added to start the reaction. The reaction was terminated by adding 200 µL 1 M Na_2_CO_3_. A calibration curve was prepared with *o-*nitrophenol concentrations ranging from 0.00625 to 1 mM. One katal (kat) of *β*-galactosidase activity was defined as the release of 1 mol *o*-nitrophenol per second.

### Mass spectrometry analysis

Mass spectrometry analysis was performed by the Mass Spectrometry Unit of the Core Facility Hohenheim at the University of Hohenheim (Stuttgart, Germany). Firstly, proteins were in-gel digested using trypsin, based on the protocol described by Shevchenko et al. [47]. After digestion, samples were dried in a vacuum centrifuge and dissolved in 0.1% TFA for nano-LC-MS/MS analysis.

Nano-LC-ESI-MS/MS experiments were done on an EASY-nLC1200 system (Thermo Fisher Scientific, Germany) coupled to a Q-Extractive HF mass spectrometer (Thermo Fisher Scientific, Germany) using a Nanospray Flex source (Thermo Fisher Scientific, Germany). Peptide separation was performed on a C18 analytical column (NanoEase M/Z HSS C18 T3, 1.8 μm 100 Å 75 μm x 250 mm column; Waters GmbH, Eschborn, Germany) at 40 °C. Gradient elution was done at a flow rate of 250 nL/min employing a solvent gradient comprising a 2–55% solution of solvent B over a 30 min period. The solvents used were 0.1% formic acid (solvent A) and 0.1% formic acid in 80% acetonitrile (solvent B). Survey spectra (m/z = 200–2000) were detected in the Orbitrap at a resolution of 60,000 at m/z = 200. Data-dependent MS/MS mass spectra were generated for the 20 most abundant peptide precursors in the Orbitrap using higher-energy collision dissociation fragmentation at a resolution of 15,000 with a normalized collision energy of 27.

Mascot 2.6 (Matrix Science, UK) was used as a search engine for protein identification. The spectra were searched against the *β*-gal-Pw protein sequence and the *Komagataella phaffii* protein database from NCBI [48]. Search parameters specified no enzyme, a 5 ppm mass tolerance for peptide precursors and 0.02 Da tolerance for fragment ions. Methionine oxidation was designated as a variable modification, while carbamidomethylation of cysteine residues was set as a fixed modification. The resulting data were then transferred to Scaffold^TM^Software 4.10.0 (Proteome Software, USA) for validation.

### Investigation of product inhibition by D-galactose

Product inhibition by D-galactose was investigated in the *β*-gal-Pw preparation and the commercial *β-*galactosidase preparations Saphera 2600L and Lactozym Pure 6500L (Novozymes, Bagsværd, Denmark), Maxilact Super (DSM, Heerlen, Netherlands), GODO-YNL2 (GODO SHUSEI Co., Ltd., Chiba, Japan) and Dairyzym Y 50 L (SternEnzym GmbH & Co. KG, Ahrensburg, Germany). For this purpose, the effect of D-galactose on *β-*galactosidase activity was investigated at 8 °C in activity buffer (100 mM potassium phosphate, 5 mM MgCl_2_, pH 6.75). The determination of *β*-galactosidase activity described above was realized at 8 °C with 100 µL substate solution (50 mM *o*NPGal) mixed with 80 µL of the activity buffer containing 350 mM D-galactose (final concentration in the assay: 140 mM). An amount of 15 µkat_*o*NPGal_/L (determined at 8 °C without D-galactose) was used for each *β*-galactosidase preparation.

### Statistical analysis

All experiments were done at least in biological duplicates with three independent measurements and evaluated by determining the standard deviation with Excel (Microsoft, Redmond, USA). Data are presented as mean values with standard deviation.

## Results

### Investigation of various signal peptides for *β*-galactosidase (*β-*gal-Pw) secretion

An attempt was firstly made to produce *P. wynnii β*-galactosidase (*β-*gal-Pw) in *K. phaffii* by secretion. Accordingly, 11 different signal peptides were tested (Additional file 1: Table S4). Among them were ten based on the *α*MF signal peptide from *Saccharomyces cerevisiae* [36] and one signal peptide derived from *K. phaffii*. The latter was the signal peptide of an endo-1,3(4)-*β*-glucanase (UniProt ID: C4QW71), natively secreted by *K. phaffii* [49, 50]. Correctly assembled P_GAP_-signal-peptide-*β-*gal-Pw cassette plasmids (Additional file 1, Fig. S2) were transformed into *K. phaffii* (integration method: Additional file 1, Fig. S3). Up to 10 clones per construct were restreaked on BMD_X−Gal_ agar plates (Additional file 1, Fig. S4). All of these clones turned blue on the BMD_X−Gal_ agar plates while the *K. phaffii* control strain without a cassette plasmid integrated appeared white and did not turn blue. The recombinant *K. phaffii* clones obtained were cultivated in honeycomb plates and investigated for *β-*gal-Pw secretion. No extracellular *β-*galactosidase activity was detected for any of the clones, indicating that none of the signal peptides tested resulted in the secretion of *β-*gal-Pw. Additionally, the clones using the *α*MF∆_no_Kex, Glucoamylase*-α*MF∆, Invertase-*α*MF∆ and Killer-*α*MF∆ signal peptides were cultivated in shake flasks (growth curves: Additional file 1, Fig. S5). Again, none of the clones showed any extracellular activity. Therefore, the clones were additionally analyzed for intracellular activity (only in the 48 h samples), which were determined to be 6.7 ± 0.8 (*α*MF∆_no_Kex), 37.1 ± 1.1 (Glucoamylase*-α*MF∆), 61.4 ± 1.1 (Invertase*-α*MF∆) and 22.3 ± 0.3 µkat_*o*NPGal_/L_culture_ (Killer*-α*MF∆).

**Fig. 1:**
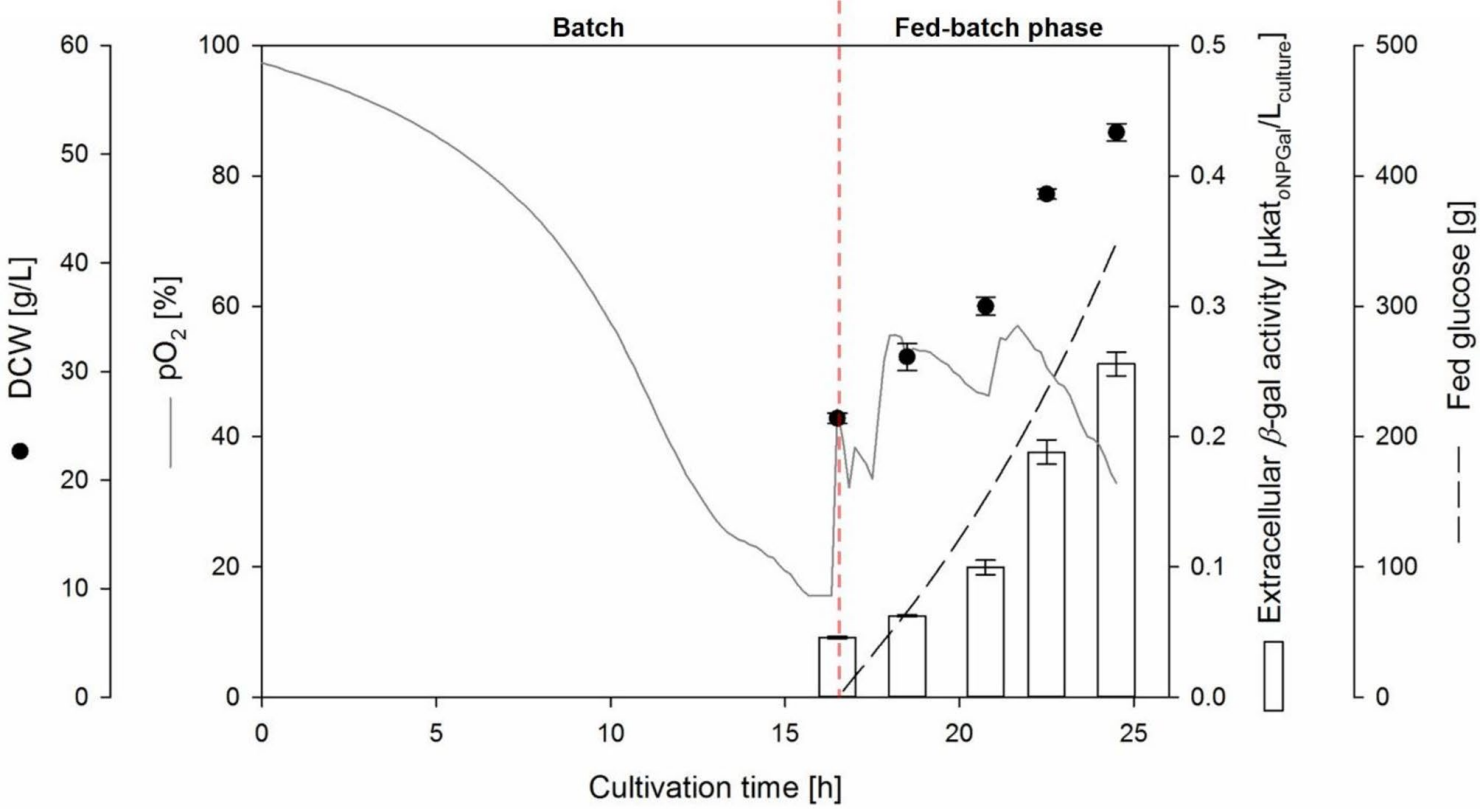
Fed-batch bioreactor cultivations of *K. phaffii* P_GAP_*-*Killer-*α*MF∆-*β*-gal-Pw. Cultivation was done in BSM_glucose_ medium at pH 6 and 30 °C with an initial fermentation volume of 3 L

Furthermore, the *K. phaffii* clone using the Killer*-α*MF∆ signal peptide was cultivated in a bioreactor under controlled conditions. After a 16.5 h batch phase, the glucose was completely consumed, as indicated by an increase in pO_2_ and verified by glucose test strips (Fig. 1). At this time, a dry cell weight (DCW) of 25.7 ± 0.5 g/L and an extracellular *β-*galactosidase activity of 0.046 ± 0.001 µkat_*o*NPGal_/L_culture_ was measured. An exponential glucose feed was executed resulting in a µ of 0.122 (Additional file 1, Fig. S6). After 8 h of feeding (24.5 h cultivation time), an extracellular *β*-galactosidase activity of 0.26 ± 0.01 µkat_*o*NPGal_/L_culture_ was determined, suggesting that apparently a low amount of *β-*gal-Pw was secreted, but that could also be derived from cell lysis. At the same time, a much higher intracellular *β-*galactosidase activity of 12.9 ± 0.5 µkat_*o*NPGal_/L_culture_ was determined (Additional file 1: Fig. S7).

Additionally, the secretome and proteome of this recombinant *K. phaffii* with the Killer-*α*MF∆ signal peptide was analyzed by SDS-PAGE and specific protein bands were analyzed for *β-*gal-Pw by mass spectrometry (Additional file 1: Fig. S8). The N-terminal part of the Killer*-α*MF∆ signal peptide for both intra- and extracellular *β-*gal-Pw was found by mass spectrometry, demonstrating that the complete signal peptide sequence was still present at the N-terminus of the *β*-gal-Pw. Based on these findings, problems in the secretory pathway of this enzyme in *K. phaffii* were suspected, and therefore the focus was shifted to the investigation of intracellular *β-*gal-Pw production in the yeast.

### Investigation of the intracellular *β*-galactosidase (*β-*gal-Pw) production

**Fig. 2:**
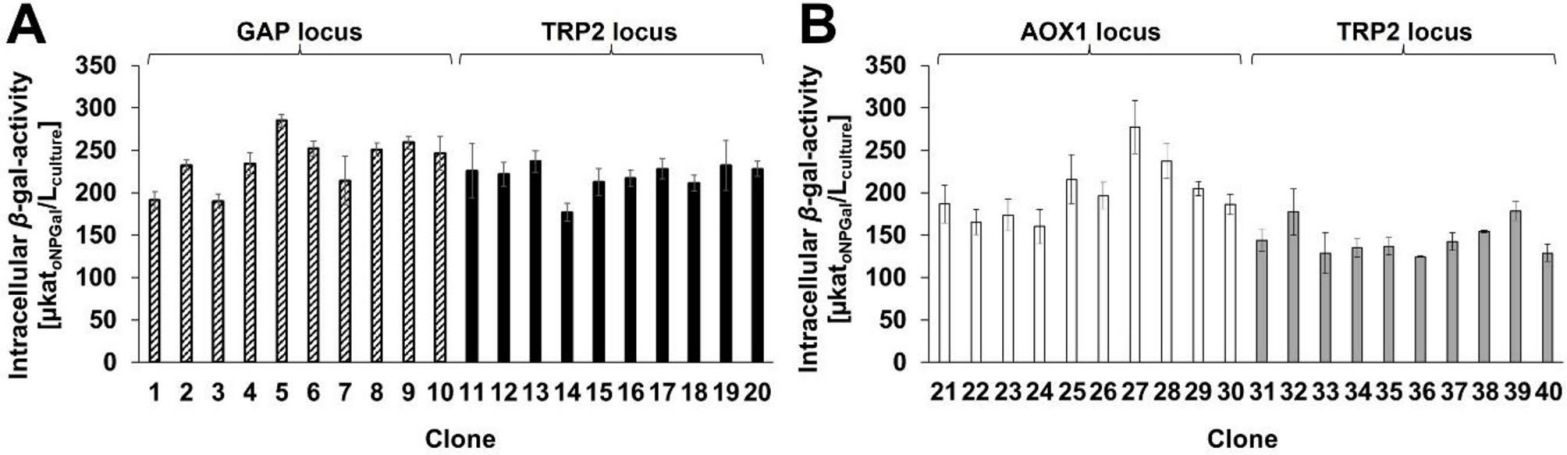
Intracellular *β*-galactosidase activities of recombinant *K. phaffii* clones with P_GAP_-*β-*gal-Pw **(A)** and P_AOX1_-*β-*gal-Pw **(B)** cassette plasmids integrated into different genomic loci (deep well plate cultivations, 500 µL, 30 °C, 48 h)

Two different promoters, the methanol inducible P_AOX1_ and the constitutive P_GAP_, were investigated for intracellular *β-*gal-Pw production. Correctly assembled cassette plasmids (Additional file 1, Fig. S9) were integrated into either one of the promoter regions P_AOX1_ or P_GAP_ by homologous recombination or the TRP2 locus by a recombinase-based method (Additional file 1: Fig. S10 and Fig. S11). A total of four different *K. phaffii* constructs were generated. Regarding any possible clonal variations [51], a microscale screening of ten clones of each construct was done to identify those *K. phaffii* clones showing the highest intracellular *β*-galactosidase activity (Fig. 2).

There was no significant difference in *β-*galactosidase activities for the P_GAP_ clones (Fig. 2A) using the GAP (mean value 235 ± 29 µkat_*o*NPGal_/L_culture_) or TRP2 locus (mean value 219 ± 16 µkat_*o*NPGal_/L_culture_). By contrast, when the P_AOX1_-*β-*gal-Pw cassette plasmid was integrated into the AOX1 locus, higher *β*-galactosidase activities were obtained (Fig. 2B) (mean value 200 ± 34 µkat_*o*NPGal_/L_culture_) than by using the TRP2 locus (mean value 145 ± 18 µkat_*o*NPGal_/L_culture_). When using P_GAP_, the highest *β-*galactosidase activity of 285 ± 7 µkat_*o*NPGal_/L_culture_ was obtained with clone 5 and the GAP locus (Fig. 2A). When using P_AOX1_, the highest *β-*galactosidase activity of 277 ± 31 µkat_*o*NPGal_/L_culture_ was obtained with clone 27 and the AOX1 locus (Fig. 2B). Therefore, these two strains were used in the following fed-batch bioreactor cultivations. The *K. phaffii* wildtype strain was checked separately and showed no endogenous *β*-galactosidase activity under the same conditions.

### Production of *β*-galactosidase (*β-*gal-Pw) in fed-batch bioreactor cultivations

Promoter-specific fed-batch bioreactor cultivations were done to determine which of the two promoter systems would achieve the highest *β-*galactosidase activities under promoter-adapted cultivation conditions. Thus, different carbon sources and feeding procedures were used depending on the promoter used for the *β-*gal-Pw expression.

### Production of *β*-galactosidase (*β-*gal-Pw) under the control of P_GAP_

**Fig. 3:**
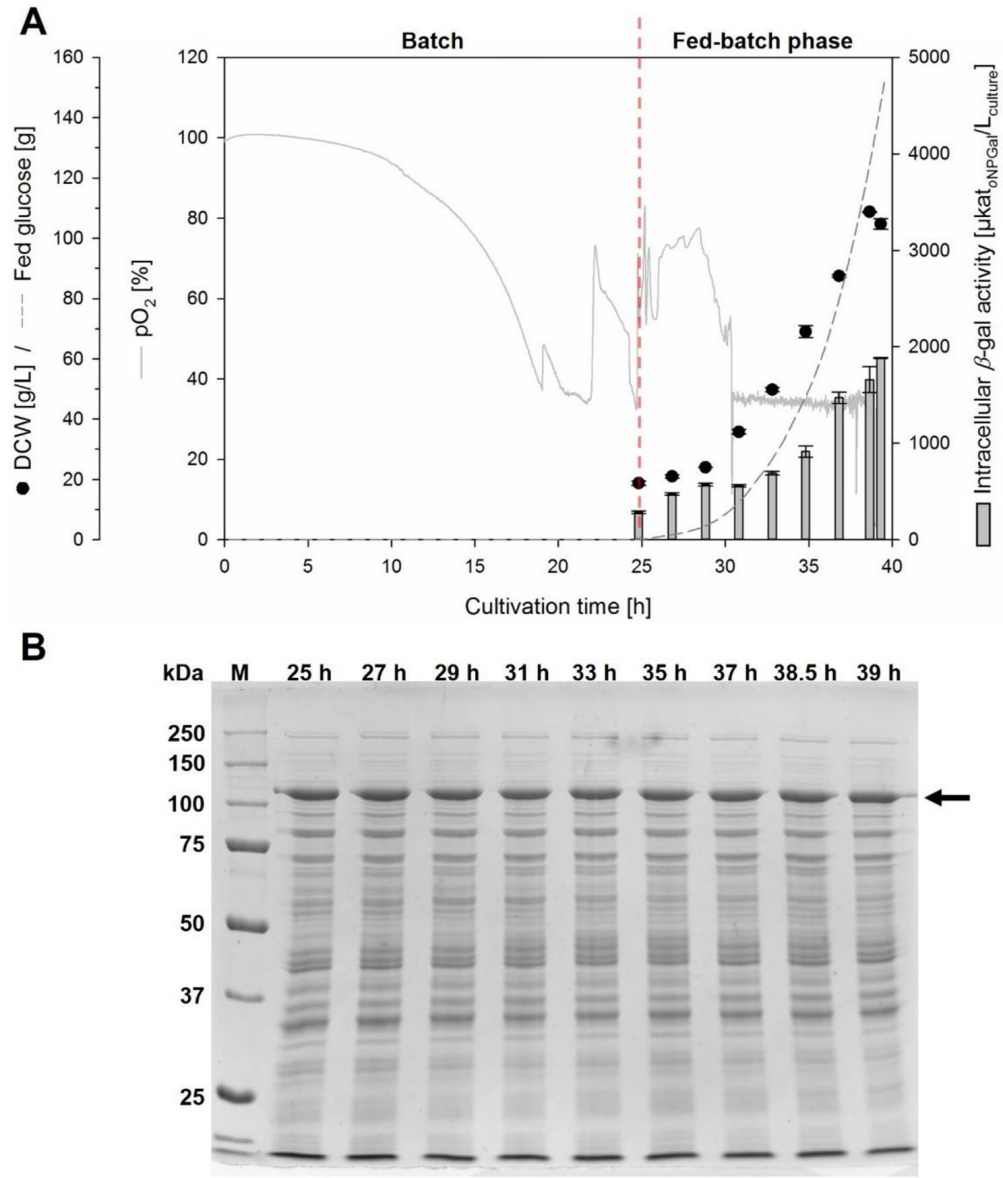
Production of *β*-gal-Pw under the control of P_GAP_ in a fed-batch bioreactor cultivation. **(A)** Cultivation of *K. phaffii* with P_GAP_*-β*-gal-Pw integrated into the GAP locus. Cultivation was done in BSM_glucose_ medium at pH 5 and 30 °C with an initial fermentation volume of 500 mL. **(B)** SDS PAGE analysis of cell-free extracts obtained from fed-batch cultivation at certain times. An amount of 5 µg protein was loaded per lane. The arrow indicates the theoretical molecular weight of *β*-gal-Pw

During cultivation of the *K. phaffii* strain (clone 5) with a P_GAP_-*β-*gal-Pw cassette plasmid integrated into the GAP locus, the carbon source glucose was completely depleted after about 25 h, as indicated by an increase in pO_2_ and verified by glucose test strips (Fig. 3A). At this time, a dry cell weight of 19 ± 1 g/L and a *β*-galactosidase activity of 285 ± 13 µkat_*o*NPGal_/L_culture_ was measured.

A glucose feed was then started (fed-batch phase). Since higher product yields are generally obtained if the specific growth rate µ is close to the maximum specific growth rate µ_max_ using P_GAP_ [26], an exponential glucose feed was applied. A µ_max_ of 0.181/h was determined in a previous batch bioreactor cultivation of the same strain (Additional file 1: Fig. S12). In order to avoid glucose accumulation during the fed-batch phase, µ was kept at values ≤ µ_max_. A µ of 0.171 ± 0.003/h was reached (Additional file 1: Fig. S13) during the fed-batch phase, resulting in a final dry cell weight of 105 ± 2 g/L (OD_600nm_ 440 ± 10) after 14.5 h of glucose feed (Fig. 3A). The cultivation was stopped at this time point as the maximum working volume of the bioreactor was reached. During the fed-batch phase, the glucose concentrations in the medium were always ≤ 0.5 g/L according to the glucose test strips used. The intracellular *β*-galactosidase activity increased similar to the biomass during the fed-batch phase. At the end of the cultivation, the highest *β*-galactosidase activity was reached with 1884 ± 6 µkat_*o*NPGal_/L_culture_ (Fig. 3A). Finally, a specific cell activity of 18.0 ± 0.1 µkat_*o*NPGal_ per gram dry cell weight was produced. Considering the cultivation time, a specific productivity *q*_p_ of 3.14 ± 0.05 µkat_*o*NPGal_/g_DCW_/h was achieved.

SDS PAGE analysis showed a distinguished protein band at approximately 120 kDa that fits to the molecular weight of *β-*gal-Pw (Fig. 3B). The intensity of this protein band did not vary over the cultivation time, which was consistent with the specific *β*-galactosidase activity, that remained almost constant at 106 ± 9 µkat_*o*NPGal_/g_protein_ throughout the cultivation.

### Production of *β*-galactosidase (*β-*gal-Pw) under the control of P_AOX1_

**Fig. 4:**
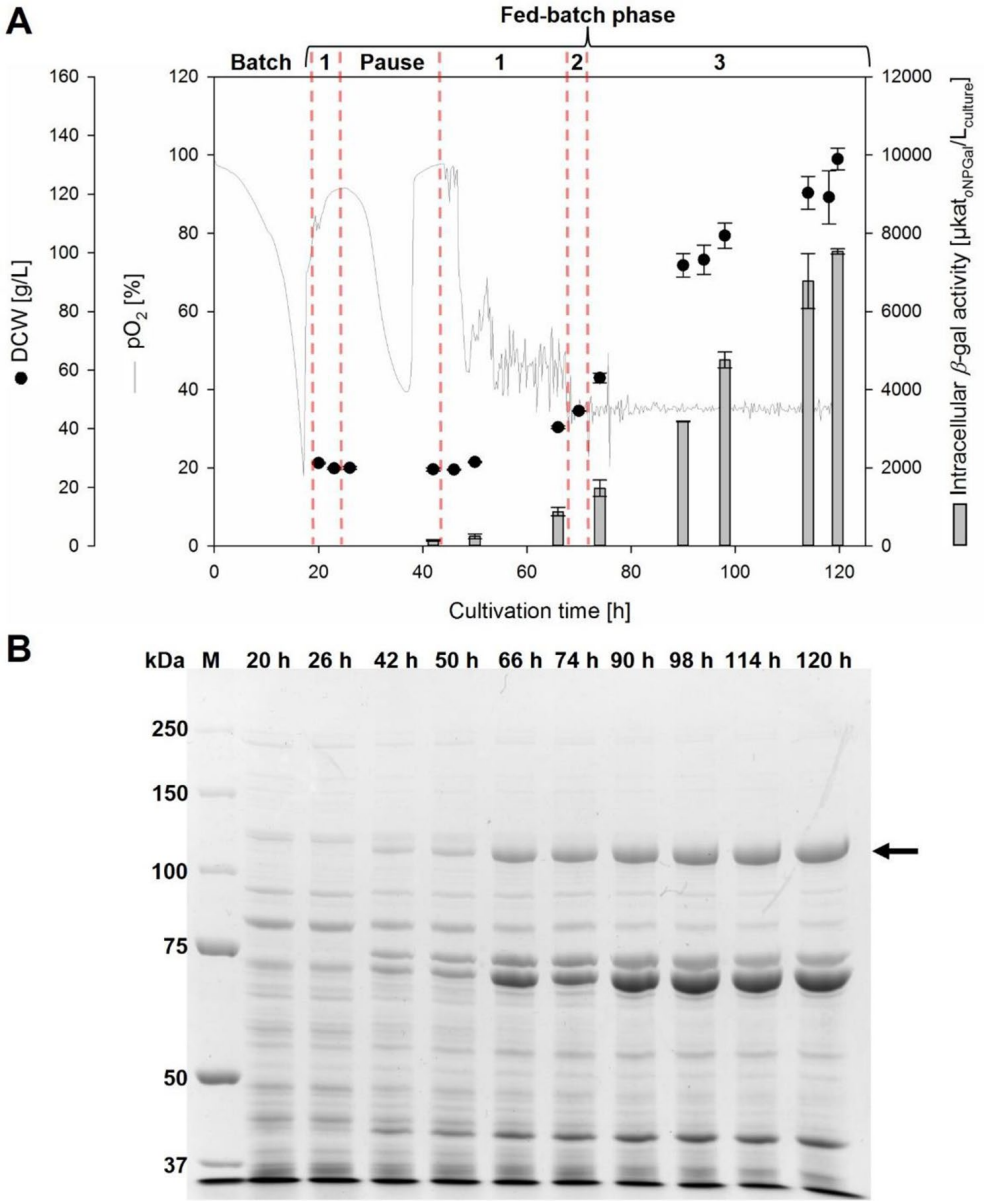
Production of *β*-gal-Pw under the control of P_AOX1_ in a fed-batch bioreactor cultivation. **(A)** Cultivation of *K. phaffii* with P_AOX1_-*β*-gal-Pw integrated into the AOX1 locus. Cultivation was done at pH 5 and 30 °C with an initial fermentation volume of 500 mL. BSM_glycerol_ medium was used during the batch phase. *β*-gal-Pw expression was induced using different methanol feed rates [mL/h]: 1 = 1.80; 2 = 3.65; and 3 = 5.45. **(B)** SDS PAGE analysis of cell free extracts obtained from fed-batch cultivation. An amount of 5 µg protein was loaded per lane. The arrow indicates the theoretical molecular weight of *β-*gal-Pw

Glycerol was used as a first carbon source in the batch phase for cultivation of the *K. phaffii* strain with a P_AOX1_-*β-*gal-Pw cassette plasmid integrated into the AOX1 locus (Fig. 4A).

After approximately 20 h, the glycerol was completely depleted, as indicated by a pO_2_ spike. Subsequently, *β-*gal-Pw expression was induced by starting the methanol feed (feed rate 1.8 mL/h; fed-batch phase). Straight from the beginning of the fed-batch phase (26 h of cultivation), methanol accumulated up to 3.77 ± 0.05% (v/v). Therefore, the feed was paused until the methanol was completely consumed. After 42 h of cultivation, the methanol feed was started again at a feed rate of 1.8 mL/h (phase 1). At this time, a *β-*galactosidase activity of 143 ± 17 µkat_*o*NPGal_/L_culture_ and a dry cell weight of 26 ± 1 g/L was determined. After 67 h of cultivation, the feed rate was increased to 3.65 mL/h for about 4 h (phase 2), followed by a further increase to the final constant methanol feed rate of 5.45 mL/h (phase 3). The methanol concentration in the medium until the end of cultivation was always below 0.1% (v/v). The biomass and *β-*galactosidase activity increased constantly till the end of the cultivation. After a total cultivation time of 120 h, the highest *β-*galactosidase activity of 7537 ± 66 µkat_*o*NPGal_/L_culture_ and a dry cell weight of 132 ± 4 g/L (OD_600nm_ 525 ± 8) was obtained (Fig. 4A), corresponding to a specific cell production of 57.1 ± 0.5 µkat_*o*NPGal_ per gram dry cell weight. A specific growth rate µ of 0.023 ± 0.001 per hour was obtained during cultivation, resulting in a specific cell productivity *q*_p_ of 1.53 ± 0.03 µkat_*o*NPGal_/g_DCW_/h.

SDS PAGE analysis showed a protein band appearing at approximately 120 kDa after 42 h of cultivation (Fig. 4B). The intensity of this protein band increased with increasing cultivation time, suggesting that it corresponded to *β-*gal-Pw. Additionally, the specific *β*-galactosidase activity increased over the cultivation time, reaching its maximum at 201 ± 7 µkat_*o*NPGal_/g_protein_ after 120 h. In addition, after 42 h of cultivation, a protein band appeared at approximately 75 kDa with increasing intensity over the cultivation time, as was also seen for the 120 kDa protein band, suggesting that it was a degradation product of the *β-*gal-Pw. Another protein band was visible just below the 75 kDa protein band (Fig. 4B). This was probably the native alcohol oxidase 1 (AOX1; Uniprot ID: F2QY27) from *K. phaffii* with a molecular weight of ∼ 74 kDa. The AOX1 can account for up to 30% of all proteins when *K. phaffii* was grown on methanol [52].

### Partial purification of the recombinant *β*-galactosidase (*β-*gal-Pw)

**Fig. 5:**
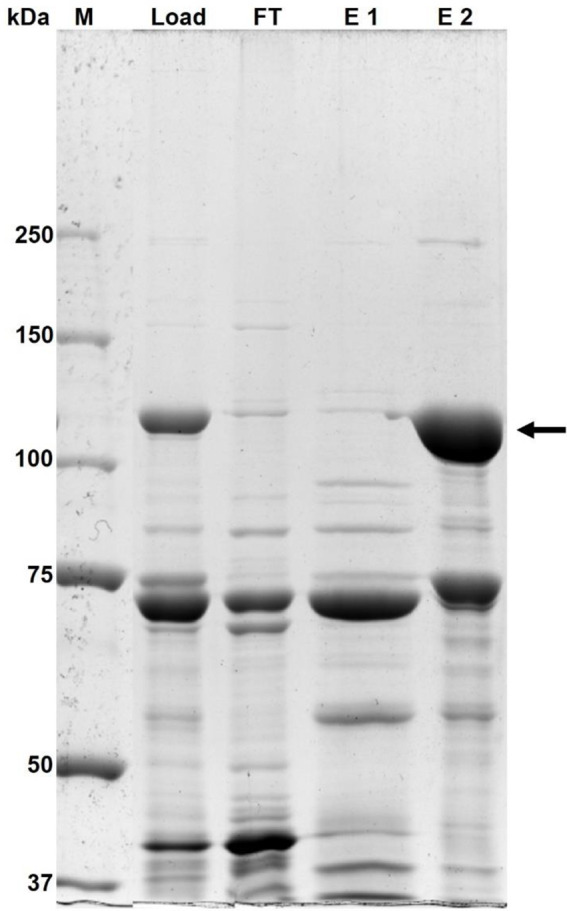
SDS PAGE analysis of the partial *β*-gal-Pw purification by hydrophobic interaction chromatography using step gradient elution. M = protein standard; Load = cell free *β-*gal-Pw preparation (20% (NH_4_)_2_SO_4_ saturation), FT = flow-through, E 1 = elution fraction 1 (20% elution buffer), E 2 = elution fraction 2 (100% elution buffer), *β-*gal active fraction. An amount of 5 µg protein was loaded per lane. The arrow indicates the theoretical molecular weight of *β*-gal-Pw

The *K. phaffii* biomass (obtained with P_AOX1_) was harvested and treated further to obtain a *β*-gal-Pw preparation that is comparable with common commercial enzyme preparations of the food industry, which are concentrated and partially purified aids for various applications. After cell disruption of 450 g of *K. phaffii* wet biomass (obtained from about 1 L cell culture) a volumetric *β*-galactosidase activity of 10799 ± 455 µkat_*o*NPGal_/L_cell free extract_ was determined in the cell free extract obtained. Afterwards, *β-*gal-Pw was partially purified by hydrophobic interaction chromatography (HIC) using a two-step elution strategy (Chromatogram: Additional file 1: Fig. S14). The protein pattern of both steps is shown in Fig. 5. The summary of the partial purification is shown in Table 1.

The first elution step E 1 (20% elution buffer) comprised native *K. phaffii* proteins (Fig. 5). The elution step E 2 (100% elution buffer) contained the recombinant *β-*gal-Pw. A prominent protein band at ∼ 120 kDa was visible in this E 2 sample on the SDS PAGE, which is consistent with the molecular weight of *β*-gal-Pw. Another prominent protein band in the E 2 sample occurred at 75 kDa, which is probably the native alcohol oxidase 1 from *K. phaffii* since the expression was regulated by the AOX1 promotor.

Finally, the *β*-gal-Pw was partially purified 4.31-fold (specific *β-*galactosidase activity of 575 ± 25 µkat_*o*NPGal_/g_protein_) with a yield of 55 ± 2% (Table 1).

**Table 1:** Partial purification of *β*-galPw

	Volume [mL]	EA_total_ [µkat_oNPGal_]	Protein_total_ [g]	EA_spec._ [µkat_oNPGal_/g_protein_]	Yield [%]	PF [-]
Cell free extract	665	7182 ± 303	53.9 ± 0.5	133 ± 6	100 ± 4	1.00 ± 0.04
(NH_4_)_2_SO_4_	675	5681 ± 50	42.5 ± 0.2	134 ± 1	79 ± 1	1.00 ± 0.01
HIC	450	3943 ± 170	6.9 ± 0.3	575 ± 25	55 ± 2	4.31 ± 0.19

EA_total_ = total *β*-gal activity; Protein_total_ = total protein content, EA_spec._ = specific *β*-gal activity, PF = purification factor

**Fig. 6:**
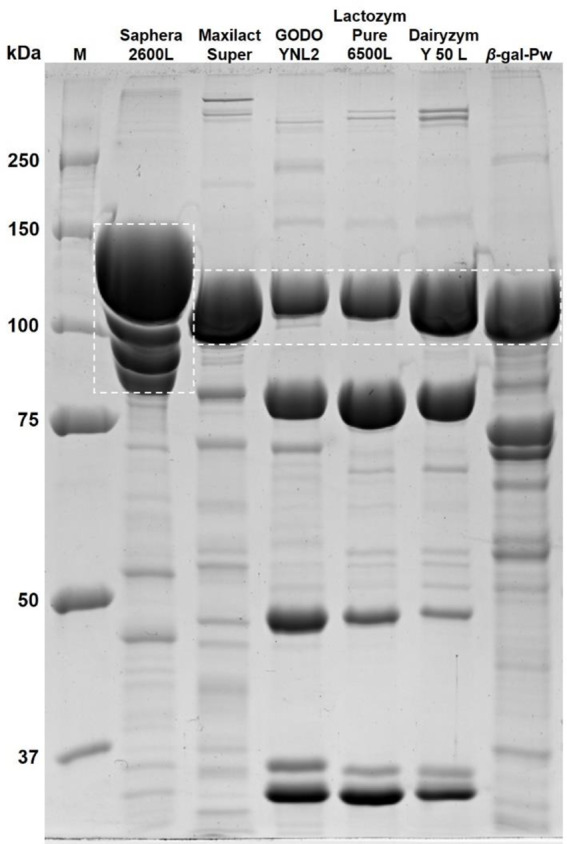
SDS PAGE analysis of various commercial *β*-galactosidase preparations and the *β*-gal-Pw preparation. M = protein standard; 5 µg protein was loaded per lane. Expected molecular weights of *β-*galactosidases are outlined with a white dashed line

After HIC, the *β-*gal-Pw sample was concentrated, and 50% (v/v) glycerol was added to extend the shelf life, as is often done with commercial enzyme preparations. Finally, 240 mL of *β-*gal-Pw enzyme preparation with a volumetric *β-*galactosidase activity of 12842 ± 407 µkat_*o*NPGal_/L (specific *β*-galactosidase activity of 509 ± 16 µkat_*o*NPGal_/g_protein_) was obtained. Thus, a final *β*-gal-Pw preparation with a total *β-*galactosidase activity of 3082 ± 98 µkat_*o*NPGal_ was obtained from 1 L recombinant *K. phaffii* culture. This *β*-gal-Pw preparation was compared with various commercially available and well-known *β*-galactosidase preparations by SDS PAGE (Fig. 6). A distinguished protein band around 100–120 kDa was seen in all preparations.

When the specific *β*-galactosidase activities were measured and compared among the *β*-galactosidase preparations, a 5–16-fold higher value was determined for the commercial preparations (Table 2). It must be mentioned that these activities were gathered with the synthetic substrate *o*NPGal and not with the natural substrate lactose.

**Table 2:** Comparison of specific activities (EA_spec._) Of different *β*-galactosidase preparations. The *β*-gal activity was determined in 100 mM potassium phosphate buffer containing 5 mM MgCl_2_ (pH 6.75) at 37 °C

*β*-galactosidase preparation	Manufacturer	EA_spec._ [µkat_oNPGal_/g_protein_]
Saphera 2600L^1^	Novozymes	2524 ± 65
Maxilact Super^2^	DSM	6050 ± 72
GODO YNL2^2^	GODO SHUSEI Co., Ltd.	6325 ± 76
Lactozym Pure 6500L^2^	Novozymes	6946 ± 210
Dairyzym Y 50 L^2^	SternEnzym GmbH & Co. KG	8031 ± 80
*β*-gal-Pw^3^	This study	509 ± 16

^1^ *Bifidobacterium bifidum*

^2^ *Kluyveromyces lactis*

^3^ *Paenibacillus wynnii*

Regarding later use in lactose hydrolysis in food matrices, however, it must be noted that the commercial *β-*galactosidases are inhibited by the generated product D-galactose, while the *β-*gal-Pw is even activated by D-galactose (Additional file 1: Fig. S15), which was also shown by Lutz-Wahl et al. [13].

## Discussion

First, extracellular production of *β*-gal-Pw was investigated. All recombinant clones showed a blue color on BMD_X−Gal_ agar plates, indicating integration of the cassette plasmids with a functional *β-*gal-Pw expression cassette. *β*-galactosidases are able to hydrolyze X-Gal resulting in the formation of a blue dye [53]. However, in small scale screening experiments as well as in shake flask cultivations no extracellular *β*-galactosidase activities could be detected, indicating problems in the secretory pathway of this enzyme. Intracellular *β-*galactosidase activity was determined for some of the *K. phaffii* clones, indicating that the *β*-gal-Pw could already fold in the cytosol and probably did not enter the secretory pathway. Furthermore, mass spectrometry analysis showed that the Killer-*α*MF∆ signal peptide was still present at the N-terminus of the active intracellular *β-*gal-Pw, indicating problems with the translocation into the ER, where processing of the signal peptide actually occurs [54]. It was shown in other studies that if the *α*MF signal peptide was fused to proteins which could already fold in the yeast cytosol, translocation across the ER membrane was not possible [55, 56]. To overcome this problem, a hybrid signal peptide consisting of the *Saccharomyces cerevisiae* Ost1 pre signal peptide fused to a variant of the *α*MF pro signal peptide was used [55, 56]. In comparison to the *α*MF pre signal peptide, which directs posttranslational translocation across the ER membrane [57], the Ost1 pre signal peptide directs co-translational translocation, thus, giving the protein no chance to fold in the cytosol [58, 59]. The signal peptides used in this study, such as the *α*MF signal peptide, are described to guide proteins into the posttranslational secretory pathway, which might be unfavorable for the secretion of large and natively intracellular proteins such as *β*-gal-Pw. Therefore, signal peptides mediating the co-translational translocation of *β*-gal-Pw across the ER membrane could possibly be more successful guiding this enzyme through the secretory pathway of *K. phaffii*.

In the bioreactor cultivation of recombinant *K. phaffii* with the Killer*-α*MF∆ signal peptide, extracellular *β-*galactosidase activity was measurable for the first time. However, mass spectrometry analysis showed that the complete signal peptide was still present at the N-terminus of the extracellularly present *β-*gal-Pw, indicating that *β*-gal-Pw was only apparently secreted, but was in fact most probably released by natural cell lysis.

With the prokaryotic organism *B. subtilis*, on the other hand, *β*-gal-Pw could be secreted using various signal peptides [17]. The highest extracellular *β*-galactosidase activity of 77.5 ± 10 µkat_*o*NPGal_/L_culture_ was achieved using the PhoD signal peptide mediating secretion via the twin-arginine translocation pathway. However, intracellular *β*-galactosidase activity of up to 19 µkat_*o*NPGal_/L_culture_ was still detected, suggesting that there may also be some limitations in the secretory pathway of *β-*gal-Pw in *B. subtilis* [17].

Secretion of *β*-gal-Pw did not work in *K. phaffii*, therefore, intracellular production of this enzyme was investigated using P_GAP_ and P_AOX1_. For both promoter systems, the *β-*gal-Pw could be produced intracellularly in high amounts of about 1900 (P_GAP_) and 7500 (P_AOX1_) µkat_*o*NPGal_/L_culture_, indicating that no problem occurred during transcription or translation but at the level of secretion.

Looser et al. reported when using P_AOX1_ that higher product yields are often obtained when the recombinant *K. phaffii* strain is grown considerably below its maximum specific growth rate µ_max_. However, when using P_GAP_, the optimum specific growth rate µ_opt_, at which production performance is best, is often near to µ_max_ [26]. Although the *K. phaffii* strain, with the *β-gal-Pw* gene under the control of P_GAP_, was cultivated nearly at its µ_max_, a four-fold higher volumetric *β-*galactosidase activity was obtained when P_AOX1_ was used. Also, a three-fold higher *β*-galactosidase activity per gram dry cell weight was obtained when P_AOX1_ was used. A difference in the *β*-galactosidase activity per gram dry cell weight may also be due to a different copy number of the expression cassette in the *K. phaffii* genome [60], which was not investigated in this study. Additionally, the promoter-specific cultivation conditions were empirically based on literature, therefore, it cannot be ruled out that the differences in activity per gram dry cell weight may have resulted from different physiological conditions influenced by the chosen cultivation conditions [61]. Further studies would need to be done to investigate this. However, higher expression levels were also observed in other studies when P_AOX1_ was used instead of P_GAP_ [62–65]. Considering the whole cultivation time, the *K. phaffii* clone using P_GAP_ for the expression of *β*-gal-Pw showed a two-fold higher specific productivity (3.14 ± 0.05 µkat_*o*NPGal_/g_DCW_/h) than the clone using P_AOX1_ (1.53 ± 0.03 µkat_*o*NPGal_/g_DCW_/h). Accordingly, multiple cultivations could also be done with the P_GAP_ clone to achieve similar *β-*galactosidase activity to the P_AOX1_ clone in a comparable amount of time.

*K. phaffii* was also investigated for the production of *β-*galactosidases from other host organisms (Table 3).

**Table 3:** Comparison of *β*-galactosidase production in *K. phaffii*

Source	Promoter	Expression	Initial working volume [L]	EA [kU_oNPGal_/ L_culture_]	Reference
*A. oryzae*	P_AOX1_ P_GAP_	extracellular extracellular	0.8 0.025	1435 n.d.	[31] [31]
*K. lactis*	P_AOX1_	extracellular	0.025	n.d.	[31]
*P. aerugineus*	P_AOX1_	extracellular	1.5	9500	[32]
*A. niger*	P_GAP_	extracellular	1.5	300	[33]
*A. nidulans*	P_GAP_	extracellular	1.5	4.2	[33]
*L. crispatus*	P_AOX1_	extracellular	2*	31	[34]
*B. circulans*	P_AOX1_	extracellular	2*	1.4	[16]
*P. wynnii*	P_GAP_ P_AOX1_	intracellular intracellular	0.5 0.5	113 452	This study This study

EA = *β*-gal activity; n.d. = not detected

*total volume of bioreactor

Sun et al. investigated the secretion of the *β-*galactosidase from *Kluyveromyces lactis* (*β-*gal-Kl) and *Aspergillus oryzae* (*β-*gal-Ao) in *K. phaffii* [31]. *β*-gal-Kl could not be secreted by *K. phaffii* using the *α*MF signal peptide and P_AOX1_. By contrast, *β*-gal-Ao could be secreted using the same conditions as for *β-*gal-Kl [31]. Native *β-*gal-Kl does not have a signal peptide at its N-terminus and is, therefore, not secreted in *K. lactis*. However, *β*-gal-Ao is also secreted in *A. oryzae* itself [3]. This suggests that the likelihood of a protein being secreted may be increased if it is also secreted in the native host. *β*-gal-Pw, which could not be secreted in this study, does not have a native signal peptide and is also, therefore, unlikely to be secreted in its native host. Sun et al. additionally tested different promoters for *β-*gal-Ao expression. When, for example, P_GAP_ was used, no enzyme activity was detected. This again shows that P_AOX1_ is often more suitable for high-level production compared to P_GAP_. The highest volumetric *β*-galactosidase activity of the *β-*gal-Ao-secreting *K. phaffii* clone was obtained with 1435 kU_*o*NPGal_/L_culture_ (approximately 2.5 g_protein_/L) by co-overexpressing the chaperone BiP [31].

The highest *β-*galactosidase activity obtained to date in *K. phaffii* culture supernatant was 9500 kU_*o*NPGal_/L_culture_ (approximately 22 g_protein_/L) with *Paecilomyces aerugineus β*-galactosidase [32]. The highest intracellular *β*-galactosidase activity obtained for *β-*gal-Pw in this study, with ∼ 452 kU_*o*NPGal_/L_culture_ (7537 ± 66 µkat_*o*NPGal_/L_culture_), was comparable to that obtained for the *Aspergillus niger β-*galactosidase that could be secreted. All *β*-galactosidases shown in Table 3 that could be secreted by *K. phaffii*, except the *Lactobacillus crispatus β-*galactosidase (*β*-gal-Lc), are also secreted in their native hosts. Furthermore, most of the *β*-galactosidases in Table 3 are active as monomers, except of *β*-gal-Kl which is active as tetramer and *β-*gal-Lc as dimer [15, 34]. Since *β-*gal-Kl could not be secreted and, as a tetramer, has a more complex structure than the other *β*-galactosidases, it can be assumed that the structural properties of the enzymes may also have an influence on the secretion process. The unique feature of *β*-gal-Lc is that it is a heterodimeric protein which could be secreted by a separate expression of the two subunits, each under the control of P_AOX1_ and fused to the *α*MF signal peptide [34]. In a previous study a monomeric form of *β-*gal-Pw was determined by size exclusion chromatography [17], but no further structural properties of this enzyme have been investigated to date. However, when comparing the amino acid sequence of *β*-gal-Pw with those of the other *β*-galactosidases, a percentage identity of 42% to *β*-gal-Lc (query cover: 85%), 33% to *β-*gal-Kl(query cover: 90%) and 24% to *Bacillus circlans β*-galactosidase (query cover: 39%), as well as no similarity to the other *β*-galactosidases mentioned in Table 3, was determined by NCBI BLAST [48]. The sequence homology to *β*-gal-Kl might suggest that structural similarities could be present in *β*-gal-Pw, which could have also led to the lack of secretion of *β*-gal-Pw, as observed for *β*-gal-Kl.

In another study, *β*-gal-Pw was produced intracellularly in *E. coli* BL21(DE3) [13]. A maximum *β*-galactosidase activity of 1350 ± 12 µkat_*o*NPGal_/L_culture_ (∼ 81 kU_*o*NPGal_/L_culture_) was obtained in a 3.5 L bioreactor cultivation after 8 h total cultivation time. When *β-*gal-Pw was produced intracellularly in *K. phaffii*, a 5.6-fold higher maximum volumetric *β-*galactosidase activity was obtained using P_AOX1_.

## Conclusion

*K. phaffii* was unable to secrete the *β*-galactosidase from *Paenibacillus wynnii* (*β*-gal-Pw). Accumulated active *β*-gal-Pw within the cell as well as an unprocessed signal peptide indicated problems in the secretory pathway. A high-level production of *β-*gal-Pw was achieved in promoter-specific fed-batch bioreactor cultivations by switching to intracellular production and using different promoters. Depending on the promoter used, either higher expression levels (P_AOX1_) or higher specific productivities (P_GAP_) could be achieved for *β-*gal-Pw. The volumetric *β-*galactosidase activity obtained in this work is the highest yet achieved for *β-*gal-Pw in a fermentation.

## Electronic supplementary material

Below is the link to the electronic supplementary material.

Supplementary Material 1:

## Data Availability

No datasets were generated or analysed during the current study.
