# Supplementary material for: Recombinant production of Paenibacillus wynnii β-galactosidase with Komagataella phaffii
Source: Microb Cell Fact. 2024 Oct 5;23:263. doi: 10.1186/s12934-024-02544-5 (PMC11452983; doi:10.1186/s12934-024-02544-5)
Supplement: Supplementary file 1 — Supplementary Material 1: [file 12934_2024_2544_MOESM1_ESM.pdf]

Additional file 1

Recombinant production of *Paenibacillus wynnii β*-galactosidase with *Komagataella phaffii*

Anna Bechtel[1], Ines Seitl[1], Eva Pross[1], Frank Hetzel[1], Mario Keutgen[1], Lutz Fischer[1]*

[1]University of Hohenheim, Institute of Food Science and Biotechnology, Department of Biotechnology and Enzyme Science, Garbenstr. 25, 70599 Stuttgart, Germany

*Corresponding author:

E-mail address: lutz.fischer@uni-hohenheim.de

Tel.: +49 711 459 22311

Keywords: *Komagataella phaffii*, *Paenibacillus wynnii*, *β*-Galactosidase, Lactose hydrolysis

Table S1: Part plasmids.

Plasmid	Type	Description/Parts	Reference
pYTK001	-	Part plasmid entry vector	[1]
pYTK002	1	ConLS (assembly connector)	[1]
pYTK047	234r	GFP dropout	[1]
pYTK072	5	ConRE (assembly connector)	[1]
pYTK080	6	ZeocinR	[1]
pYTK084	8	KanR-ColE1	[1]
pPTK001	2	P_{AOX1}	[2]
pPTK002	2	P_{GAP}	[2]
pPTK005	3a	*α*MF	[2]
pPTK006	3a	*α*MF_no_EAEA	[2]
pPTK007	3a	*α*MFΔ	[2]
pPTK008	3a	*α*MFΔ_no_Kex	[2]
pPTK009	3a	*α*-Amylase-*α*MFΔ	[2]
pPTK010	3a	Glucoamylase-*α*MFΔ	[2]
pPTK011	3a	SA-*α*MFΔ	[2]
pPTK012	3a	Inulinase-*α*MFΔ	[2]
pPTK013	3a	Invertase-*α*MFΔ	[2]
pPTK014	3a	Killer-*α*MFΔ	[2]
pPTK019	4	tAOX1	[2]
pPTK020	7	*attB* (BxbI recognition site)	[2]
pPTK022	3a	signal peptide of endo-1,3(4)-*β*-glucanase (UniProt ID: C4QW71) in pYTK001	This work
pPTK023	3b	Codon optimized *β-gal-Pw* gene with overhangs for extracellular production in pYTK001	This work
pPTK024	3	Codon optimized *β-gal-Pw* gene with overhangs for intracellular production in pYTK001	This work

Table S2: Cassette plasmids constructed in this study.

Plasmid	Consisting of parts from part plasmids
GFP dropout	pYTK002, pYTK047, pYTK072, pYTK080, pYTK084, pPTK020
**For extracellular production of *β*-gal-Pw**	
P_{GAP}-*α*MF-*β*-gal-Pw	pYTK002, pYTK072, pYTK080, pYTK084, pPTK002, pPTK005, pPTK019, pPTK020, pPTK023
P_{GAP}-*α*MF_no_EAEA-*β*-gal-Pw	pYTK002, pYTK072, pYTK080, pYTK084, pPTK002, pPTK006, pPTK019, pPTK020, pPTK023
P_{GAP}-*α*MFΔ-*β*-gal-Pw	pYTK002, pYTK072, pYTK080, pYTK084, pPTK002, pPTK007, pPTK019, pPTK020, pPTK023
P_{GAP}-*α*MFΔ_no_Kex-*β*-gal-Pw	pYTK002, pYTK072, pYTK080, pYTK084, pPTK002, pPTK008, pPTK019, pPTK020, pPTK023
P_{GAP}-*α*-Amylase-*α*MFΔ-*β*-gal-Pw	pYTK002, pYTK072, pYTK080, pYTK084, pPTK002, pPTK009, pPTK019, pPTK020, pPTK023
P_{GAP}-Glucoamylase-*α*MFΔ-*β*-gal-Pw	pYTK002, pYTK072, pYTK080, pYTK084, pPTK002, pPTK010, pPTK019, pPTK020, pPTK023
P_{GAP}-SA-*α*MFΔ-*β*-gal-Pw	pYTK002, pYTK072, pYTK080, pYTK084, pPTK002, pPTK011, pPTK019, pPTK020, pPTK023
P_{GAP}-Inulinase-*α*MFΔ-*β*-gal-Pw	pYTK002, pYTK072, pYTK080, pYTK084, pPTK002, pPTK012, pPTK019, pPTK020, pPTK023
P_{GAP}-Invertase-*α*MFΔ-*β*-gal-Pw	pYTK002, pYTK072, pYTK080, pYTK084, pPTK002, pPTK013, pPTK019, pPTK020, pPTK023
P_{GAP}-Killer-*α*MFΔ-*β*-gal-Pw	pYTK002, pYTK072, pYTK080, pYTK084, pPTK002, pPTK014, pPTK019, pPTK020, pPTK023
P_{GAP}-C4QW71-*β*-gal-Pw	pYTK002, pYTK072, pYTK080, pYTK084, pPTK002, pPTK019, pPTK020, pPTK022, pPTK023
**For intracellular production of *β*-gal-Pw**	
P_{GAP}-*β*-gal-Pw	pYTK002, pYTK072, pYTK080, pYTK084, pPTK002, pPTK019, pPTK020, pPTK024
P_{AOX1}-*β*-gal-Pw	pYTK002, pYTK072, pYTK080, pYTK084, pPTK001, pPTK019, pPTK020, pPTK024

Table S3: Primer sequences.

Primer	Sequence [5´ - 3´]
C4QW71-fw	GCATCGTCTCATCGGTCTCATATGTCATTCTCTTCCAACGTGCCAC
C4QW71-rev	ATGCCGTCTCAGGTCTCAAGAACCTCCACTGACTATATTGGTCAACAG
β-gal-Pw-3b-fw	GCATCGTCTCATCGGTCTCATTCTCGTAAGAAGCTGGTCTACTCC
β-gal-Pw-3-fw	GCATCGTCTCATCGGTCTCATATGCGTAAGAAGCTGGTCTACTCC
β-gal-Pw-rev	ATGCCGTCTCAGGTCTCAGGATTTAAACCATTCTGATGGTGAAACG
TRP2-fw	AGTACCACGGTTGATCG
TRP2-rev	TCGCTTTGGGGAACATG
ConLS-rev	GCTCTACCATCTAGATGCG
GAP-fw	GGCCTACTAGACTCTCTG
tAOX1-rev	GCAAATGGCATTCTGACATCC
AOX1-fw	GACATTTGGATTTGGTTGACTC
5’-*β*-gal-Pw-rev	CATCTGCCAATGAGATGGAAC

```
atgcgtaagaagctggtctactccccaccaactaacggttacccagaatggaacaacaacccagagtgcttccagatcaa
cagaatggatgctcacgctacctggattccattcaacactactgaggacgctttgctgggtgacccacaatcttctccaa
actacttgtccttgaacggtatgtggaagttcgcttacgctgagactccagaccagagaatcagaaacttcttcgagaag
aactacgactgttcctcctgggctgaattgaacgttccatctcattggcagatgcacggttacgattacccacagtacac
caacgttagatacccatggtctgaaagagagccagagttgaagccaccatttgctccaactcagtacaacccagttggtt
cctacgttagaaccttctctgttccagaagattggtccggtaagcccgtgttcatttctttccagggtgttgaatccgcc
ttctacgtttggttgaacggtgagttggttggttactccgaggacacttttactccagctgagttcgacttgaccccata
cttgattccaggtgacaacaagttggccgtcgaggtttacagatggtgtgatgcttcttggttggaggaccaggactttt
ggagactgtccggtatcttcagagatgtctacctgtacactactccagaggctcacatctacgatttcttcgtcagaacc
gaactggacgagcagtacagacatgctgagttgcaattggacgtcaagctgatggactacttcgaaagaaccgctgaggc
tgttatcgttcacgctcagttgtacgatcacgaccagaacgctattttcgaccagccattgtctcagaccgtctacttta
actccgcttccactcaaaccttgcagttctcctcctccattatcgacccattgaagtggtctgctgagcacccaaacttg
tacaccttggttttgtccttgcaccacgtcgacggtgaattgatggaagctgtttcctgtagagtcggtttcagaacctt
cgagttgaaggacggtttgatgaagatcaacggtaagaggatcgtgttcaagggtgtcaacagacacgagttctcttgtg
acactggtagatccattgacgtcgacgacatggtcagagacatcttgttgatgaaggcccacaacatcaacgccgtcaga
acttctcactacccaaaccagactatctggtacgacttgtgtgaccagtacggtctgtacgttatcgacgagactaactt
ggaaactcacggttcttggtcctacggtcaaactgatcttggtggtaacactgtcccaggttcaagaccagaatggcgtg
ctaacgttttggacagatgcaactccatgctgcagagagacaagaaccatccatccatcgttatctggtccttgggtaac
gaatctttcggtggtgacaacttcgttgctatgcacgacttcttgaagaaagaggacccatccagactggttcactacga
aggtttgttccactacagagagtctgacgttgcttccgacattgagtccactatgtacatttccccagctgacgttgagc
agtacgctttgaacgatccaaagaaaccctacatcctgtgcgaatactctcacgccatgggtaactcttgtggtggattg
cacttgtactgggaagtgttcgagaagtacgacatcttgcagggtgctttcatttgggactggattgaccaggccatcag
attgaaacaagctgacggttctatgcacatggcctacggtggtgattttggtgaatctccacacgacggtaacttttgcg
gtaacggtctgattttcgccgacagatccgtttctccaaagctgtacgaggtcaagaagtgctaccagaacgttaagttc
gaggccgttgacttggagagaggtatctacagagttaccaaccagaacctgttcactgacttggctgaatacgctttggc
ttgggaagtttcctgcaacggtaacccagttttgaagggtactgttgaccttgctgttccagctggtgaatccgctgaaa
tttccgttccagttgttgacgagccaaacttgcaatctgaaggtgagcacgttctgacattctccctgcagttgaagaag
tccactctttgggctgatgctggtcatgaagttgcttgggagcaattcttgttgccaactccacaattcatggccggtca
agaccaagactctgttttgacttctgacagaggtgtcatcgttgaggaacaggctggtagattgactgttcaagctgctg
acgtttccctgcaattctctacttcttccggttacctgatctccatgcagaacaagggtaaagagttgctgttggagcca
gtcagaccaaacttttggcgtgctgttactgacaacgacctgggtaacaagcaccatgagagatgtgctgtttggaacac
tgctggtgctggttgtactttggcttctttcgagtcccacaagaacgttgacggtgttactgttagagccaagtacaccg
ttccaactgttccaatctccagcttgatcttggagtacagaatccaagagaacggttccatcgaggtgttcgaagaattg
tctccaggtatgggtttgccagagattccagagatcggtctgatgttcattgtcgaggacagattggataccgtgtcctg
gtatggtagaggtccacacgaaaactactgggacagaaagactggtgccagattgggttacttctctggttctgttcagg
accagttcgtcccttacattagacctcaagagtgcggtaacaagaccgacgttagattcgcttccattaccggtggtatt
aacggttccggattcagagttgatggtgaccctgttttggagttgaacgctttgccatggacaccagctgaattggaagc
taacgaccacatctacaagttgccagcttccaacaagactgtcgtcagagtcaactacaagcagatgggtgttggtggtg
ataactcttggggagctactactcacccagagtttactttgccagctgaccagacttacggtttccgtttcaccatcaga
atggtttaa
```

Fig. S1. Codon optimized *β-gal-Pw* gene sequence for *K. phaffii*.

Table S4: Signal peptides used in this study. Pre-sequences are underlined. In case of signal peptides fused to *α*MFΔ, the source refers to the pre-sequence.

Signal peptide	Source	Amino acid sequence
*α*MF[1]	*Saccharomyces cerevisiae*	MRFPSIFTAVLFAASSALAAPVNTTTEDETAQIPAEAVIGYSDLEGDFDVAVLPFSNSTNNGLLFINTTIASIAAKEEGVSLEKREAEA
*α*MF_no_EAEA[1]	*Saccharomyces cerevisiae*	MRFPSIFTAVLFAASSALAAPVNTTTEDETAQIPAEAVIGYSDLEGDFDVAVLPFSNSTNNGLLFINTTIASIAAKEEGVSLEKR
*α*MFΔ[1]	*Saccharomyces cerevisiae*	MRFPSIFTAVLFAASSALAAPVNTTTEDELEGDFDVAVLPFSASIAAKEEGVSLEKR
*α*MFΔ_no_Kex[1]	*Saccharomyces cerevisiae*	MRFPSIFTAVLFAASSALAMRFPSIFTAVLFAASSALAAPVNTTTEDELEGDFDVAVLPFSASITAKEEGVSLEKR
α-Amylase-*α*MFΔ[1]	*Aspergillus niger*	MVAWWSLFLYGLQVAAPALAMRFPSIFTAVLFAASSALAAPVNTTTEDELEGDFDVAVLPFSASIAAKEEGVSLEKR
Glucoamylase-*α*MFΔ[1]	*Aspergillus awamori*	MSFRSLLALSGLVCSGLAMRFPSIFTAVLFAASSALAAPVNTTTEDELEGDFDVAVLPFSASIAAKEEGVSLEKR
SA-*α*MFΔ[1]	*Homo sapiens*	MKWVTFISLLFLFSSAYSMRFPSIFTAVLFASSSALAAPVNTTTEDELEGDFDVAVLPFSASIAAKEEGVSLEKR
Inulinase-*α*MFΔ[1]	*Kluyveromyces lactis*	MKLAYSLLLPLAGVSAMRFPSIFTAVLFAASSALAAPVNTTTEDELEGDFDVAVLPFSASIAAKEEGVSLEKR
Invertase-*α*MFΔ[1]	*Saccharomyces cerevisiae*	MLLQAFLFLLAGFAAKISAMRFPSIFTAVLFAASSALAAPVNTTTEDELEGDFDVAVLPFSASIAAKEEGVSLEKR
Killer-*α*MFΔ[1]	*Saccharomyces cerevisiae*	MTKPTQVLVRSVSILFFITLLHLVVAMRFPSIFTAVLFAASSALAAPVNTTTEDELEGDFDVAVLPFSASIAAKEEGVSLEKR
C4QW71[2]	*Komagataella phaffii*	MSFSSNVPQLFLLLVLLTNIVSG

[1]MoClo *Pichia* Toolkit [2]
[2]This work

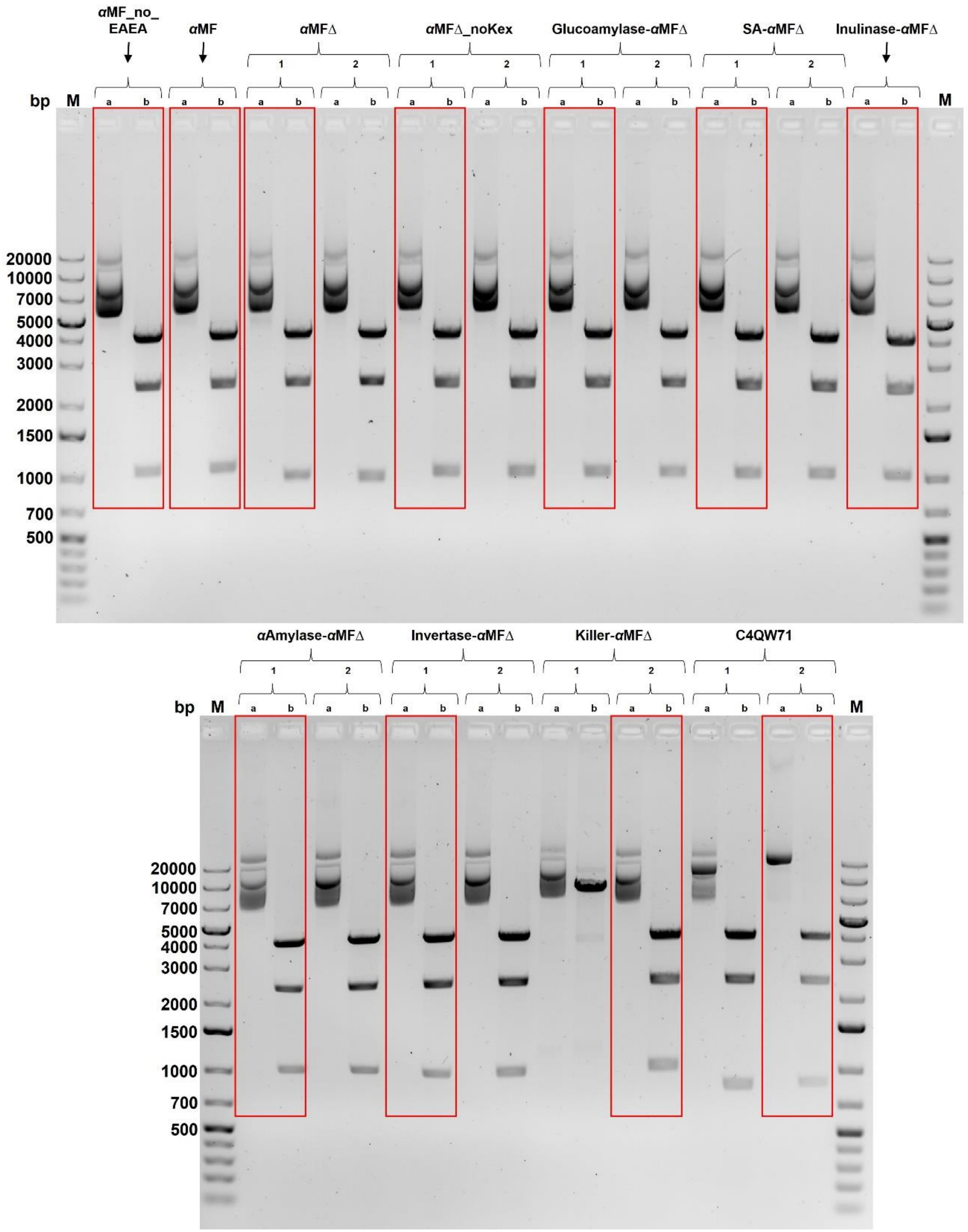

Fig. S2. Verification of correct P_{GAP}-signal-peptide-*β*-gal-Pw cassette plasmid assembly by restriction digestion using *Bam*HI and *Mfe*I. 1% (w/v) agarose gels. M = GeneRuler 1 kb Plus DNA Ladder, a = undigested, b = digested. One to two samples per construct were investigated. All samples showed expected band patterns for correct assembly except of sample 1 with the Killer-*α*MFΔ signal peptide. Red-framed samples were used for integration into the *K. phaffii* genome.

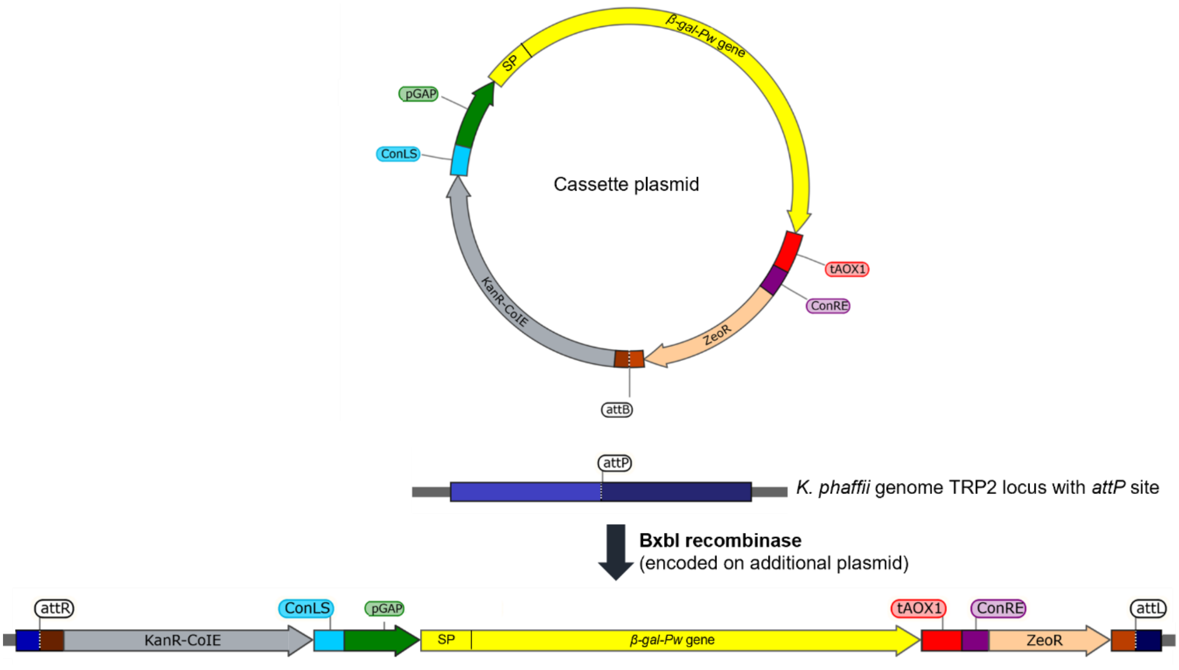

Fig. S3. Method used to integrate the cassette plasmids for extracellular *β*-gal-Pw production into the *K. phaffii* genome. SP = signal peptide.

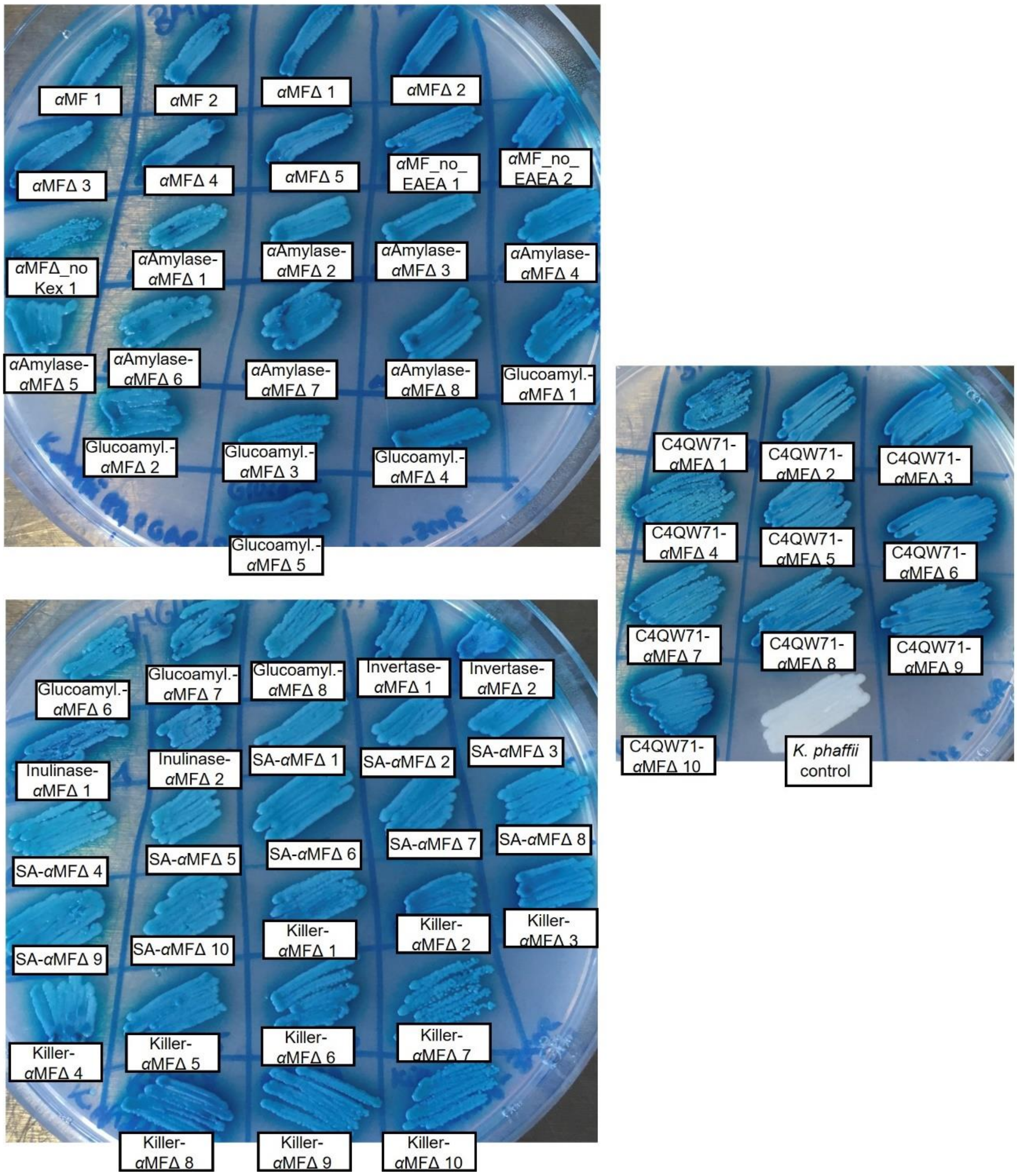

Fig. S4. Restreak of *K. phaffii* transformed with P_{GAP}-signal-peptide-*β*-gal-Pw cassette plasmids on BMD$_{X\text{-}Gal}$ agar plates. For each signal peptide used up to 10 different *K. phaffii* clones were investigated. As a control the *K. phaffii* strain with integrated PP74 vector but without integrated cassette plasmid was used. Agar plates were incubated for about 2 days at 30 °C.

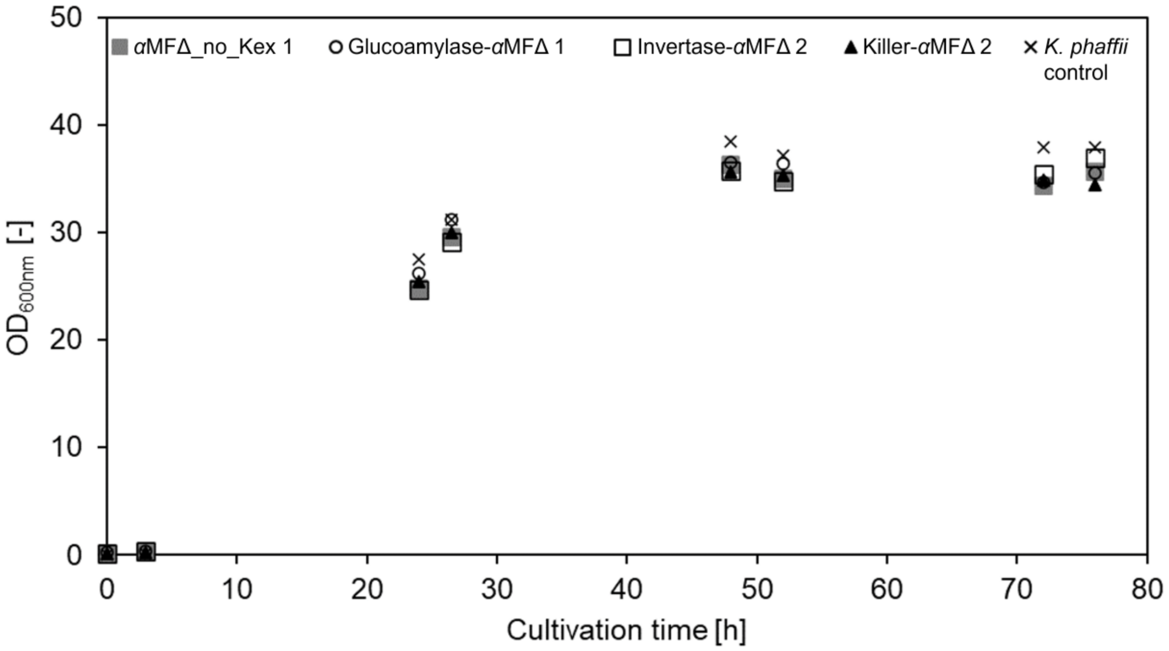

Fig. S5. Shake flask cultivation of *K. phaffii* P$_{GAP}$-signal-peptide-*β*-gal-Pw clones. *K. phaffii* clones with signal peptides *α*MFΔ_no_Kex clone 1, Glucoamylase-*α*MFΔ clone 1, Invertase-*α*MFΔ clone 2 and Killer-*α*MFΔ clone 2, previously investigated on BMD$_{X\text{-}Gal}$ agar plates (Fig. S4), were used. As a control the *K. phaffii* strain with integrated PP74 vector but without integrated cassette plasmid was used. Cultivation was done in YPD medium at 30 °C with a working volume of 100 mL.

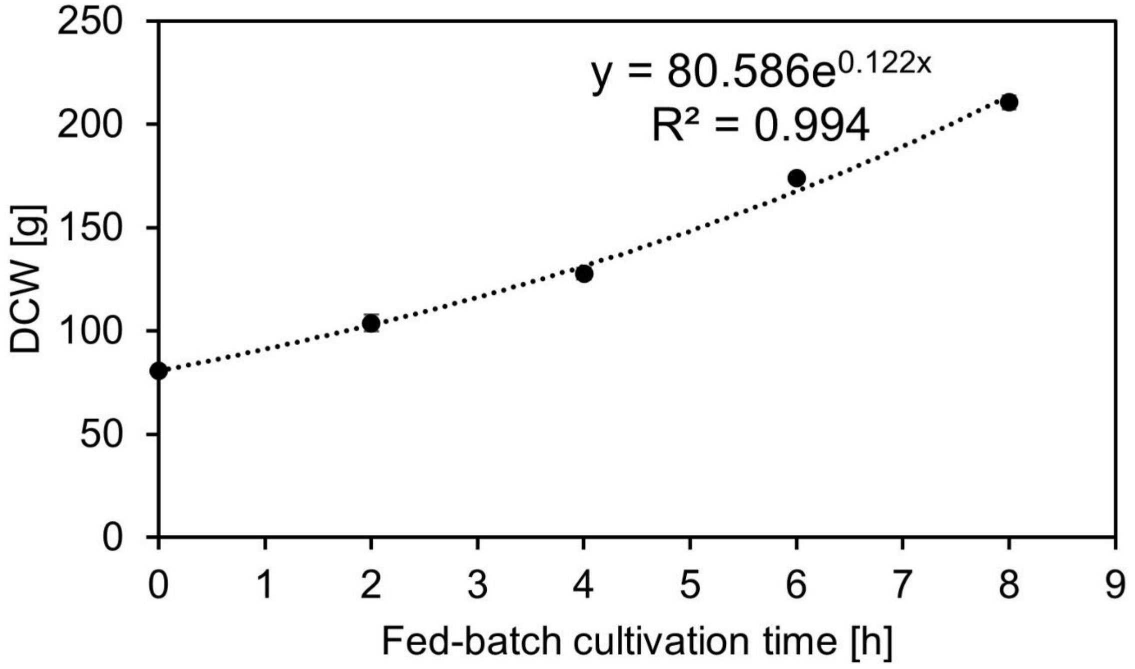

Fig. S6. Total dry cell weight during fed-batch bioreactor cultivations of *K. phaffii* P_{GAP}-Killer-*α*MFΔ-*β*-gal-Pw. Cultivation was done in $BSM_{glucose}$ medium at pH 6 and 30 °C with an initial fermentation volume of 3 L. µ was determined by fitting an exponential curve through experimental data.

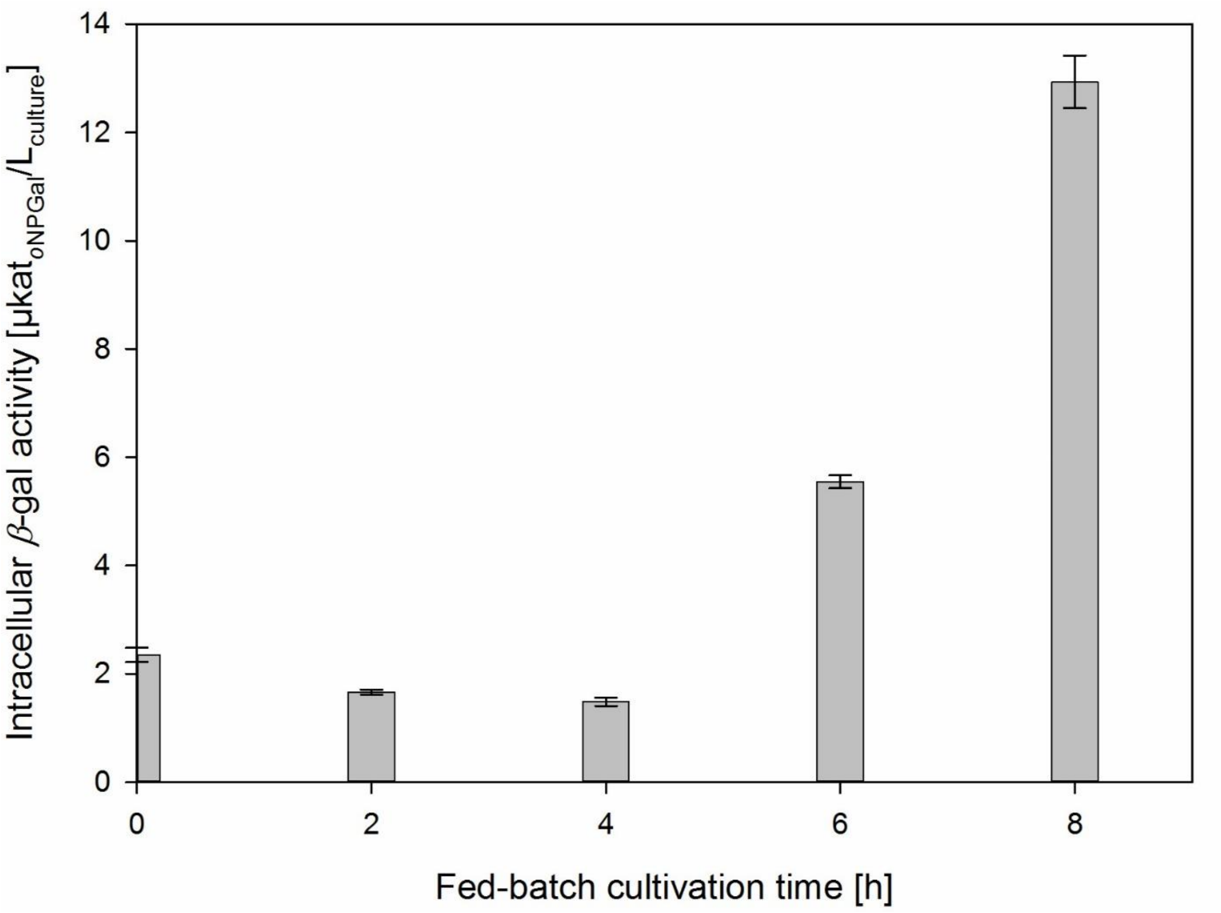

Fig. S7. Intracellular *β*-galactosidase activity during fed-batch bioreactor cultivations of *K. phaffii* P$_{GAP}$-Killer-*α*MFΔ-*β*-gal-Pw. Cultivation was done in BSM$_{glucose}$ medium at pH 6 and 30 °C with an initial fermentation volume of 3 L.

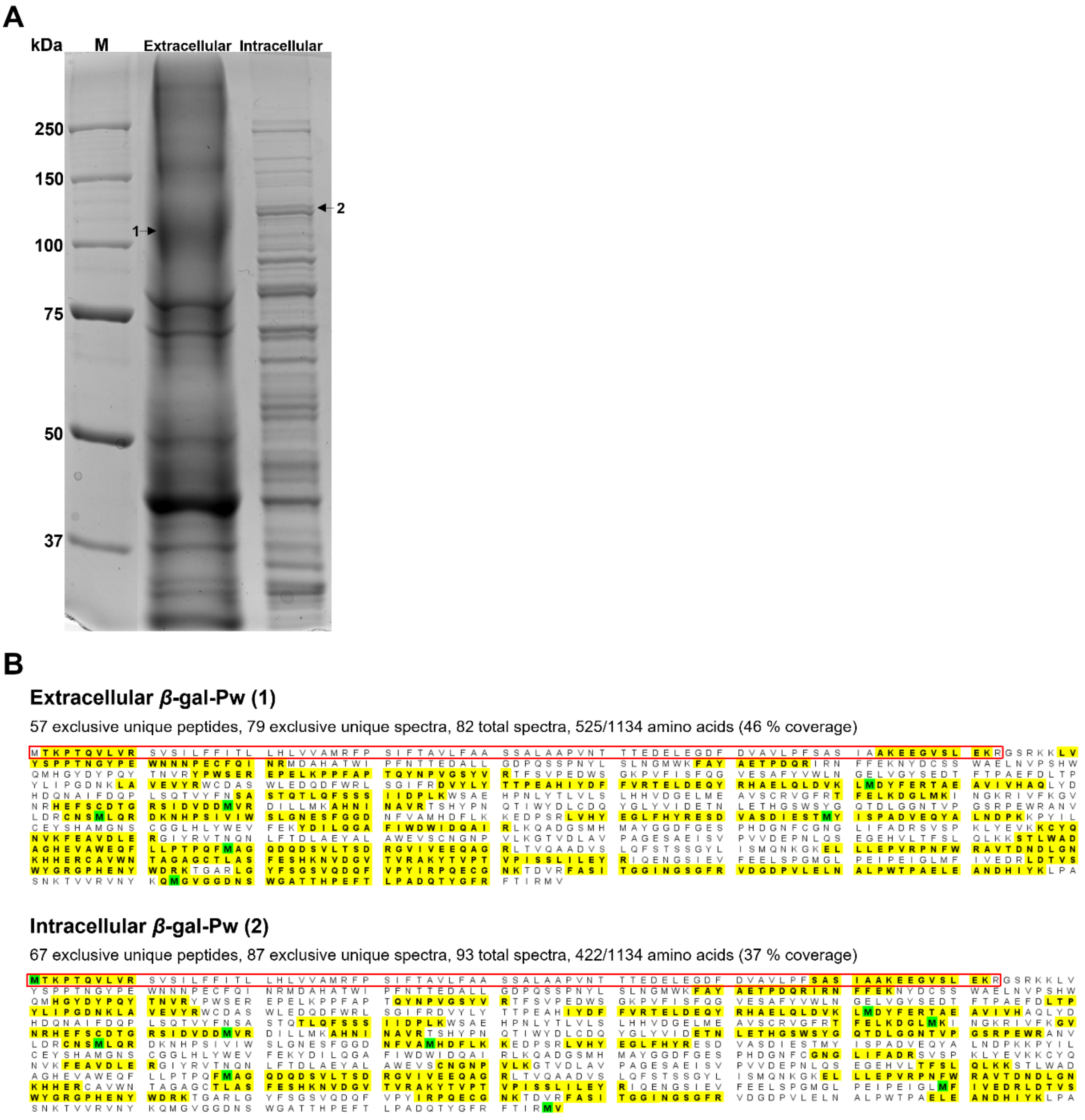

Fig. S8. Investigation of the secretome and proteome of *K. phaffii* P$_{GAP}$-Killer-*α*MFΔ-*β*-gal-Pw. (A) SDS PAGE of samples taken after 24.5 h cultivation time of fed-batch bioreactor cultivation. Extracellular: cell-free culture supernatant 160-fold concentrated; Intracellular: 5 µg protein of cell-free extract. Sample 1 and 2 were analyzed by mass spectrometry (theoretical molecular weight of *β*-gal-Pw: 120 kDa). (B) Amino acid sequence blast of *β*-gal-Pw analyzed by mass spectrometry**.** Sequences of peptides identified are highlighted in yellow, modifications in green. The Killer-*α*MFΔ signal peptide sequence is framed in red.

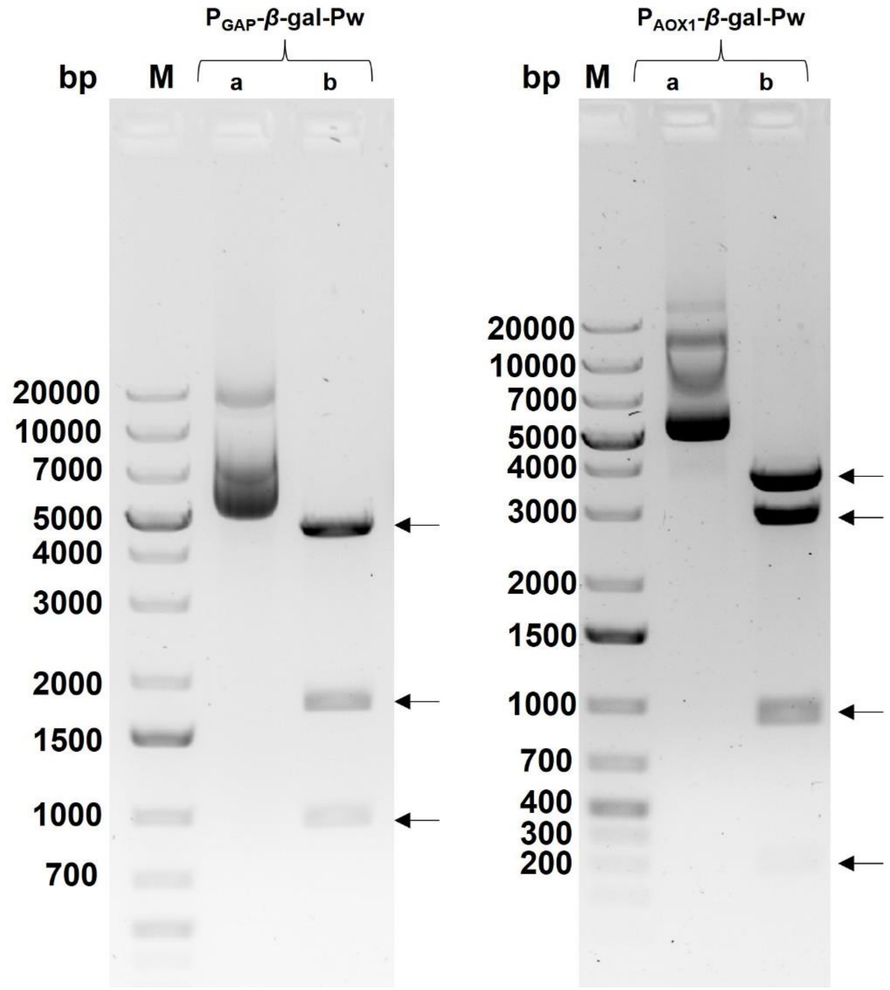

Fig. S9. Verification of correct cassette plasmid assembly by restriction digestion using *Sma*I and *Xcm*I for P_{GAP}-*β*-gal-Pw and for P_{AOX1}-*β*-gal-Pw *Pme*I and *Xcm*I. 1% (w/v) agarose gels. M = GeneRuler 1 kb Plus DNA Ladder, a = undigested, b = digested. Both samples showed expected band patterns for correct assembly (arrows indicate expected DNA bands in the digested sample).

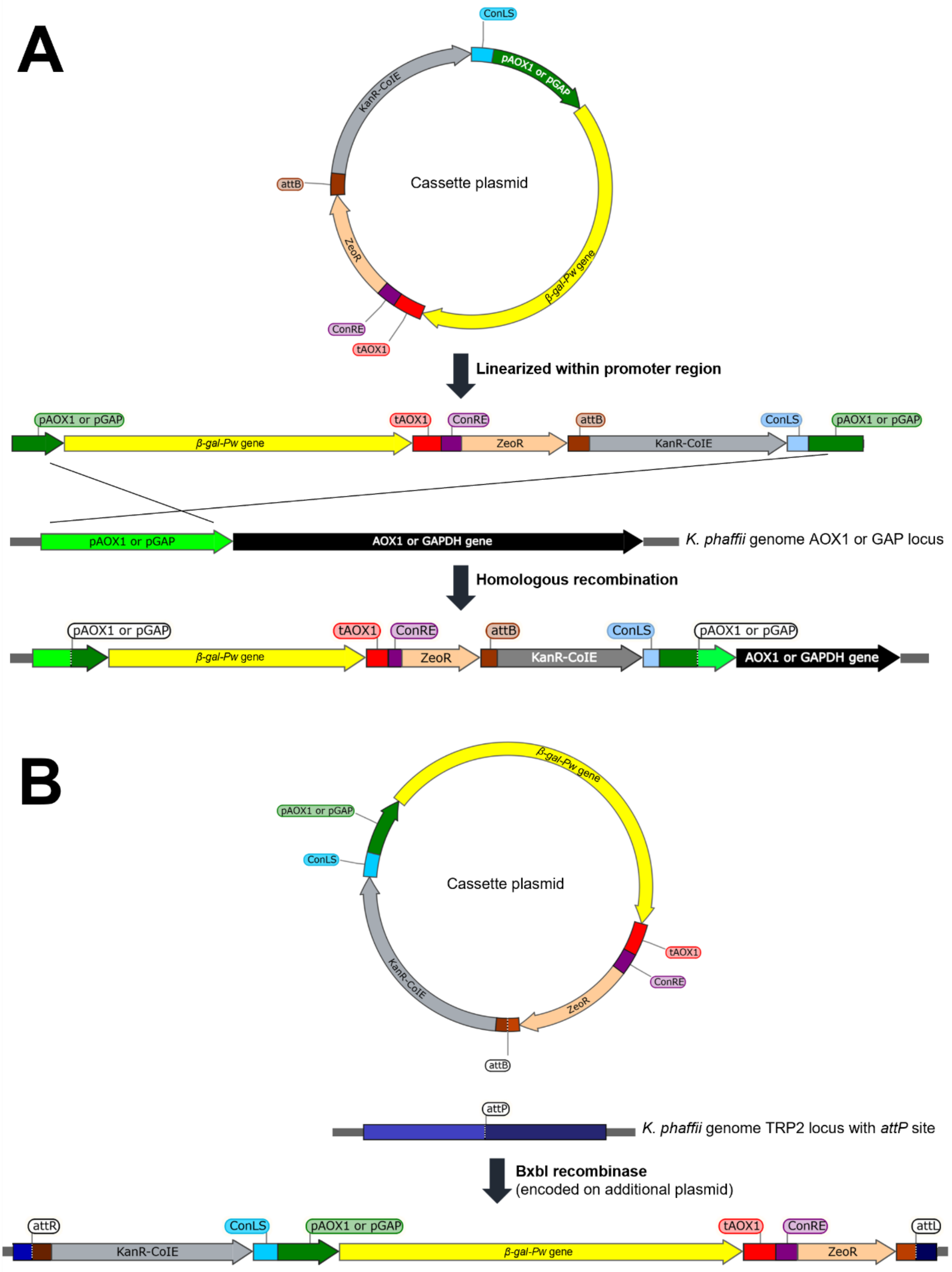

Fig. S10. Methods used to integrate the cassette plasmids for intracellular *β*-gal-Pw production into the *K. phaffii* genome. (A) Homologous recombination; (B) BxbI recombinase catalyzed site-specific recombination.

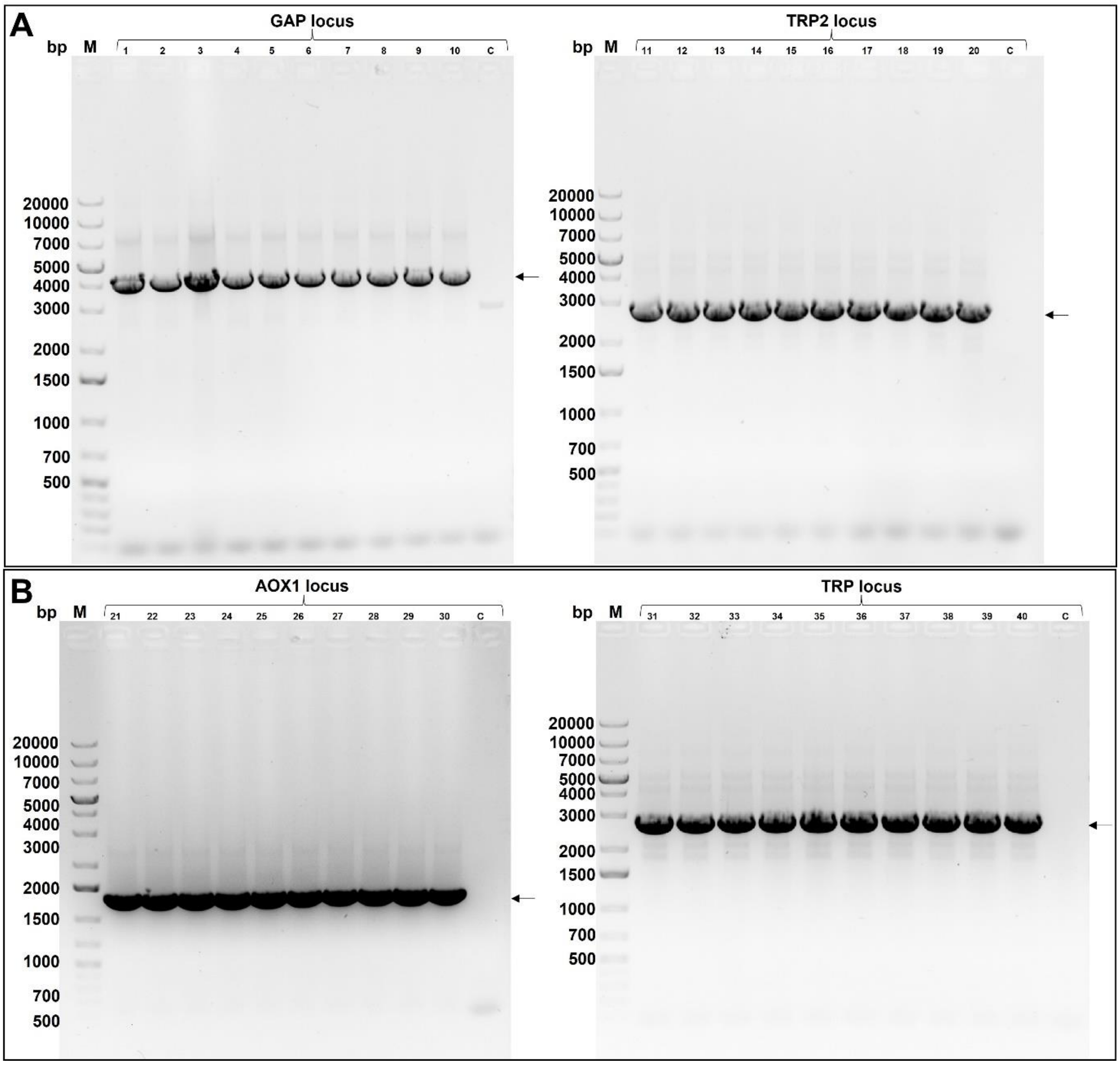

Fig. S11. Verification of the integration of P_{GAP}-*β*-gal-Pw (A) and P_{AOX1}-*β*-gal-Pw (B) cassette plasmids into different genomic loci of *K. phaffii* by PCR. 1 % (w/v) agarose gels. M = GeneRuler 1 kb Plus DNA Ladder. C = control (for GAP and AOX1 locus: *K. phaffii* ATCC 76273 wildtype strain; for TRP2 locus: *K. phaffii* with integrated PP74 vector but without integrated cassette plasmid). Arrows indicate the expected DNA bands for integration of the cassette plasmids into the respective locus.

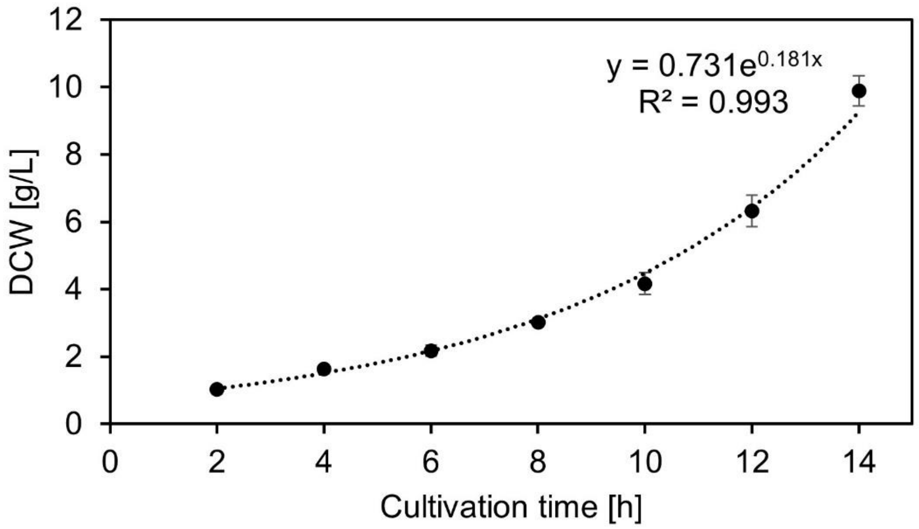

Fig. S12. Determination of μ_{max} of *K. phaffii* with P_{GAP}-*β*-gal-Pw integrated into the GAP locus. Strain was cultivated in $BSM_{glucose}$ medium at pH 5 and 30 °C with a constant working volume of 800 mL in 1 L bioreactor. μ_{max} was determined by fitting an exponential curve through experimental data.

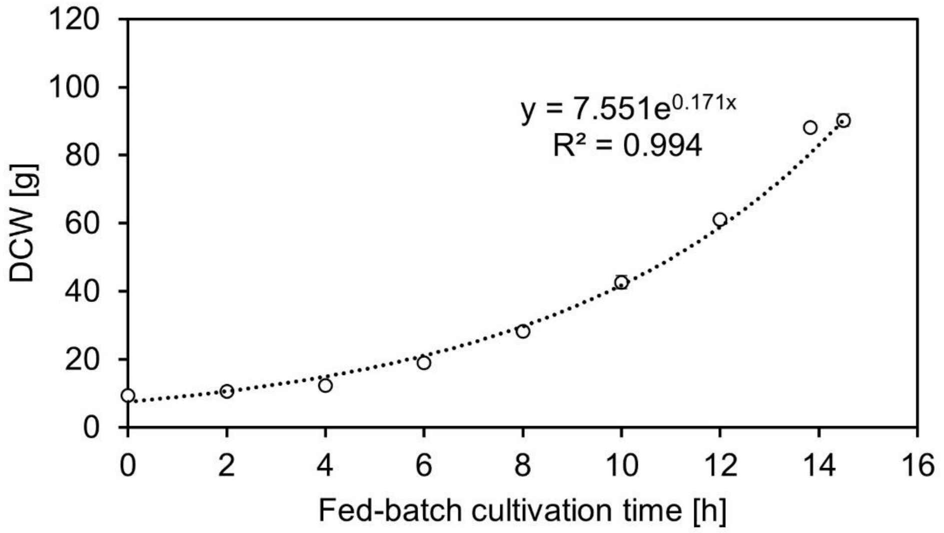

Fig. S13. Total dry cell weight during production of *β*-gal-Pw under the control of P_{GAP} in a fed-batch bioreactor cultivation. Cultivation was done in $BSM_{glucose}$ medium at pH 5 and 30 °C with an initial fermentation volume of 500 mL. µ was determined by fitting an exponential curve through experimental data.

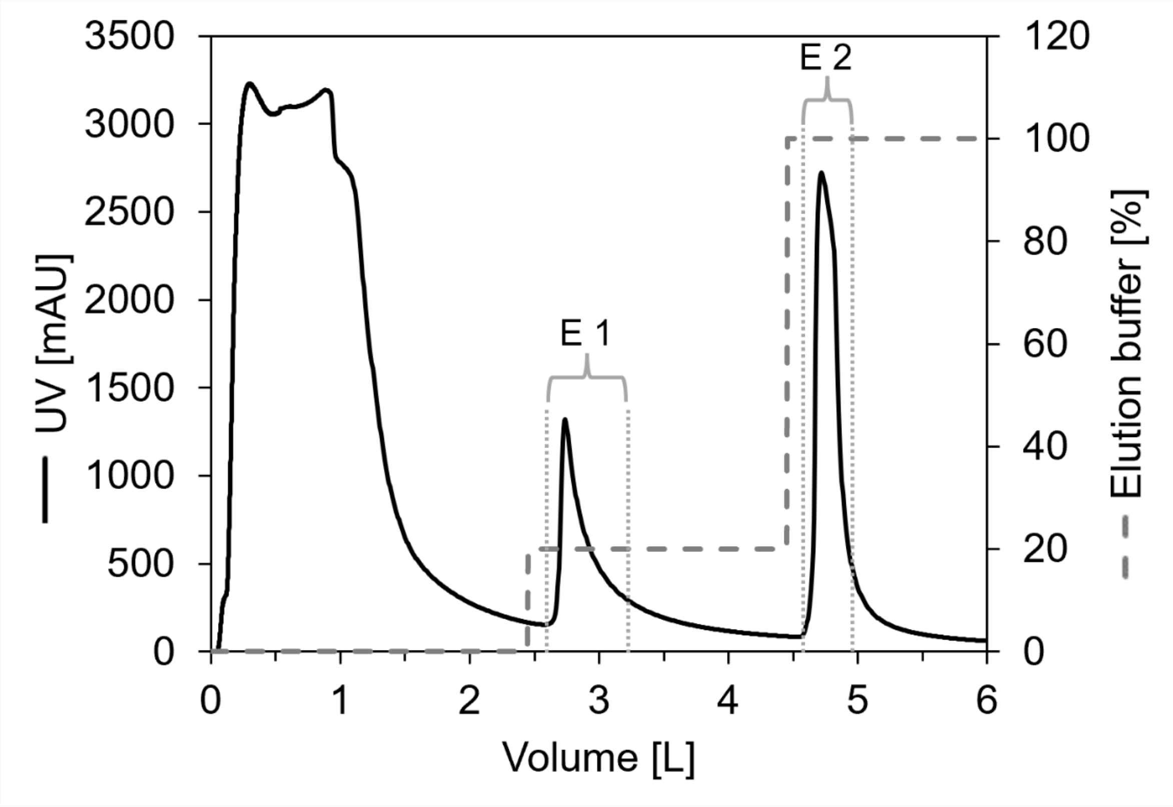

Fig. S14. Hydrophobic interaction chromatography purification of *β*-gal-Pw. Column material: Toyopearl Phenyl-650M; Column volume: 400 mL. E 1 = elution fraction 1; E 2 = elution fraction 2 (*β*-gal active fraction). Binding buffer: 100 mM potassium phosphate buffer containing 0.86 M $(NH_4)_2SO_4$ (pH 6.75). Elution buffer: 100 mM potassium phosphate buffer containing 5 mM $MgCl_2$ (pH 6.75).

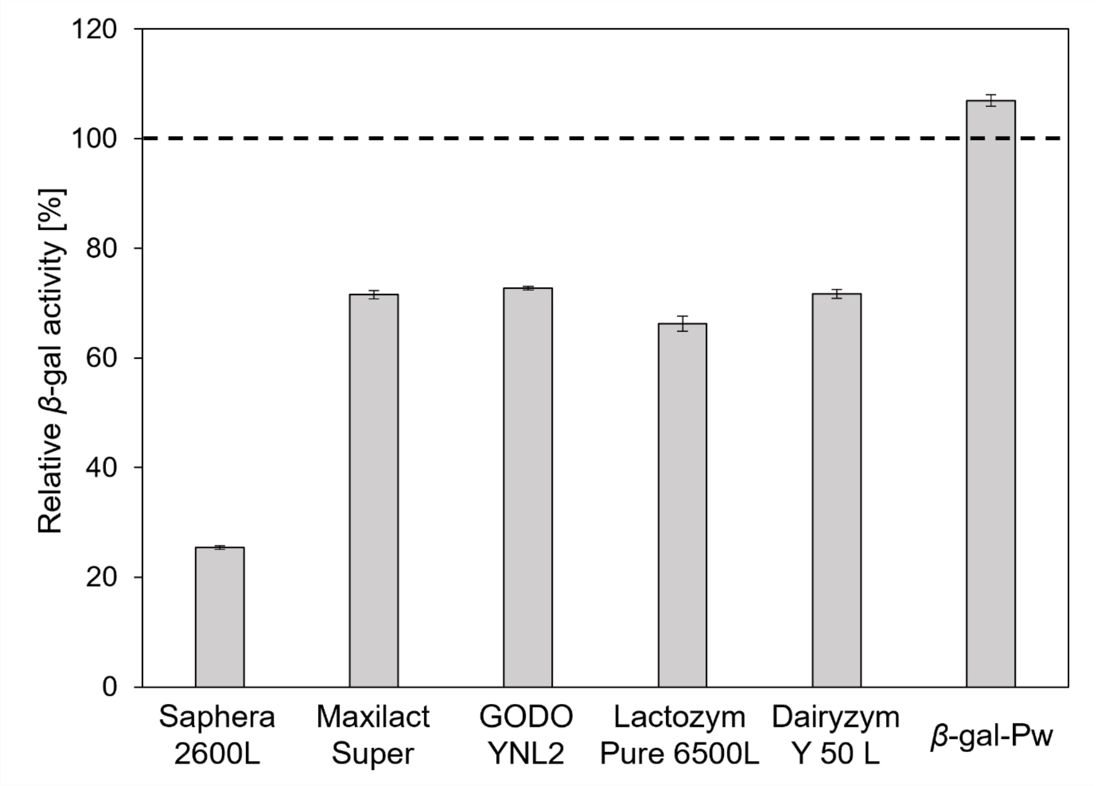

Fig. S15. Investigation of the product inhibition by D-galactose (140 mM) for various commercial *β*-galactosidases and *β*-gal-Pw at 8 °C (100 mM potassium phosphate, 5 mM $MgCl_2$, pH 6.5). The 100 % *β*-gal-Pw activity was determined without any galactose, corresponding to 15 ± 1 μkat_{oNPGal}/L.

Additional references

1. Lee ME, DeLoache WC, Cervantes B, Dueber JE. A highly characterized yeast toolkit for modular, multipart assembly. ACS Synth Biol. 2015;4:975–86. https://doi.org/10.1021/sb500366v

2. Obst U, Lu TK, Sieber V. A modular toolkit for generating *Pichia pastoris* secretion libraries. ACS Synth Biol. 2017;6:1016–25. https://doi.org/10.1021/acssynbio.6b00337
